# Interplay between nuclear survivin and the PRC2 complex and its impact on H3K27me3-directed transcriptional repression

**DOI:** 10.1242/jcs.264572

**Published:** 2026-03-17

**Authors:** Adesh D. Vaidya, Alexander J. Fezovich, Sally P. Wheatley

**Affiliations:** School of Life Sciences, University of Nottingham, Nottingham, NG7 2UH, UK

**Keywords:** Polycomb repressor complex 2, Survivin, Hypoxia, Stem cells, H3K27me3, Transcription

## Abstract

Polycomb repressor complex 2 (PRC2) tri-methylates histone 3 at lysine 27 (H3K27me3), a post translational modification that induces heterochromatin formation and transcriptional repression. Survivin (also known as BIRC5) is a nucleocytoplasmic shuttling protein that is kept out of the nucleus in clement conditions, but that accumulates there in times of stress and in certain specialised cells. Although the cytoplasmic functions of survivin are well documented, there is comparatively less understanding of its roles within the nucleus. Here, we investigated whether nuclear survivin can affect transcriptional programming. Using interaction analyses and qPCR, we report that it binds to the enzymatic subunit of PRC2 (EZH2) and H3K27me3, and causes depression of its target genes in a variety of human cells.

## Teaser

**Summary:** When the essential protein survivin is present in the nucleus of human cells it can derepress key genes that are epigenetically silenced by PRC2-mediated trimethylation of histone 3 at K27.

## INTRODUCTION

Polycomb repressor complex 2 (PRC2) is a key transcriptional regulator that catalyses tri-methylation of histone 3 at lysine 27 (H3K27me3), leading to heterochromatin formation and gene repression (Cao et al., 2002; Margueron and Reinberg, 2011). PRC2 comprises three subunits: the catalytic subunit, enhancer of Zeste (EZH2), which is a histone methyltransferase; suppressor of Zeste (SUZ) 12, which provides structural integrity to the complex; and embryonic ectoderm development protein (EED), which mediates allosteric activation of PRC2 (Kuzmichev et al., 2002; Shi et al., 2017). PRC2 is highly expressed in stem cells, and many of the downstream genes that it represses are involved in differentiation.

Survivin (also known as BIRC5) has been well documented as a chromatin-associated protein during mitosis when it is part of the chromosomal passenger complex (CPC) (Ruchaud et al., 2007; Wheatley and Altieri, 2019). In this capacity it targets aurora B kinase, to histone 3 that has been phosphorylated at threonine 3 (H3T3ph) by haspin kinase (Ruchaud et al., 2007; Kelly et al., 2010; Du et al., 2012; Jeyaprakash et al., 2011). Centromeric targeting of the CPC by survivin enables aurora-B to phosphorylate MCAK (also known as KIF2C), which ultimately ensures correct chromosome attachments to the mitotic spindle are made prior to anaphase onset (Andrews et al., 2004). This essential mechanism guarantees accurate chromosome segregation and genomic stability from one cell generation to the next (Ruchaud et al., 2007; Wheatley and Altieri, 2019). Survivin expression is normally cell cycle regulated, peaking in G2 and M phase and absent in interphase. However, this periodicity can be overridden when cells are stressed. For example, in hypoxia, the survivin gene, *BIRC5*, is activated by the transcription factor HIF1a (Peng et al., 2006). Some studies, including our own, have reported that survivin needs to be cytoplasmic to carry out its other well-known role as an inhibitor of apoptosis (Li et al., 1998), although whether this is true in all circumstances is unclear. Cells normally make a great effort to expel survivin from the nucleus, which it does via the CRM1 (exportin) pathway, which recognises a centrally placed nuclear export sequence (NES) in survivin (Rodríguez et al., 2002; Stauber et al., 2006; Colnaghi et al., 2006). Importantly, most, if not all, cancer cells constitutively overexpress survivin, and in some it is predominantly nuclear, rather than cytoplasmic (Skagias et al., 2009; Rexhepaj et al., 2010; Qi et al., 2010; Felisiak-Golabek et al., 2011; Hmeljak et al., 2011; Qian et al., 2011; Kim et al., 2012; Xie et al., 2012; Bongiovanni et al., 2012; Marioni et al., 2012; Chaopotong et al., 2012; Hu et al., 2013; Kitamura et al., 2013). Recent chromatin immunoprecipitation sequencing (ChIP-seq) data has revealed that nuclear survivin binds to 13704 genomic sites in CD4+ T-cells (Erlandsson et al., 2022). Interestingly, the majority of these sites were found to overlap with regions enriched in H3K27me3 (Erlandsson et al., 2022). This observation, together with the knowledge that survivin interacts with modified H3 during mitosis, led us to hypothesise that survivin could act as a transcriptional co-factor and alter PRC2-mediated gene expression.

The data herein presented show for the first time that survivin interacts with EZH2 and H3K27me3 in a variety of human cells. Furthermore, we report that loss of survivin leads to increased H3K27 methylation and decreased production of gene transcripts normally repressed by PRC2. To our knowledge this is the first study to offer mechanistic insight into how nuclear survivin can impact chromatin remodelling and gene expression.

## RESULTS

### Hypoxia causes nuclear accumulation of survivin

**Fig. 1.**
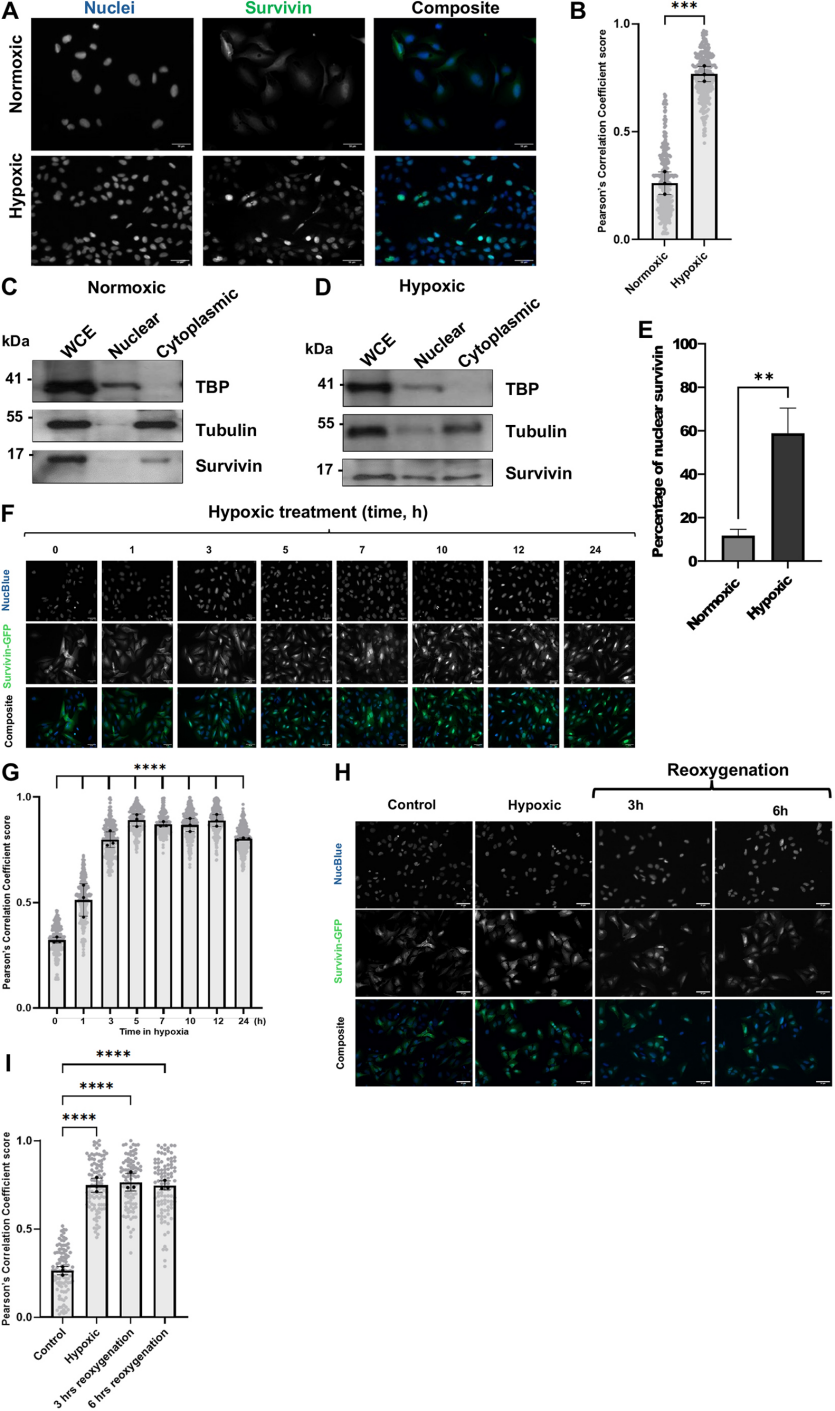
**Survivin is nuclear in hypoxic cells.** (A) U2OS cells were subjected to hypoxia (1% O_2_) for 24 h hypoxia then fixed and stained to highlight endogenous survivin (green). Nuclei were stained with NucBlue (blue). Scale bar: 50 µm. (B) Graph illustrating colocalisation analysis using Pearson's correlation coefficient score, based on the signals in A. The results are expressed as mean±s.d. from three independent experiments with *n*=250 cells. ****P<0.0001 (unpaired two-tailed Student's *t*-test). (C,D) Immunoblot analysis of U2OS cell extracts subjected to fractionation. Nuclear and cytoplasmic fractions are indicated by positive anti-TBP and tubulin bands, respectively. Survivin was more abundant in the nuclear fraction under hypoxic conditions. (E) Quantification of percentage of nuclear survivin in normoxic versus hypoxic samples using total survivin protein. Results are mean±s.d., *n*=3. **P=0.0025 (unpaired two-tailed Student's *t*-test). (F) Time-point analysis of U2OS cells expressing survivin–GFP (green) upon exposure to hypoxia. Cells were fixed and stained with NucBlue (blue) then imaged. Scale bars: 50 µm. (G) Colocalisation analysis of images in F revealed significant differences in Pearson's correlation coefficient scores when cells were subjected to hypoxia at all time intervals as compared to 0 h, with maximum nuclear expression achieved in 3 h. Results are mean±s.d., *n*=300 cells ****P<0.0001 (one-way ANOVA followed by Dunnett's post hoc test post test). (H) U2OS cells expressing survivin–GFP that had been exposed to hypoxia for 24 h were returned to normoxia for 3 or 6 h. Over this time frame survivin was retained in the nucleus. Control is normoxia. Scale bars: 50 µm. (I) Quantification of data shown in H. Results are mean±s.d., *n*=100 cells in each of three biological repeats. ****P<0.0001 (one-way ANOVA with Dunnett's post hoc test).

To illustrate that survivin becomes nuclear under stress, we exposed U2OS, HeLa and MRC5 cells to hypoxia for 24 h (Fig. 1A; Fig. S1). A clear shift from cytoplasmic predominance to nuclear localization was seen after immunostaining with anti-survivin antibodies (green), as evidenced by colocalization with NucBlue, a DNA specific dye (blue) and quantified by determining the Pearson correlation coefficient (Fig. 1B), in which a score of 1 is full nuclear localisation and 0 is no nuclear localisation.

Nuclear localisation of survivin was confirmed by immunoblot analysis of cell lysates subjected to fractionation following normoxic and hypoxic treatments (Fig. 1C,D). Here, antibodies against TATA-box binding protein (TBP) and tubulin were used to determine the purity of the nuclear and cytoplasmic fractions, respectively, as well as any impurities in the other fractions. Consistent with the imaging data in normoxic samples, survivin was more prevalent in the cytoplasmic extract and less so in the nuclear fraction (Fig. 1C,E), whereas in the hypoxic sample survivin was increased in the nuclear fraction (Fig. 1D,E).

Next, to ascertain how rapidly survivin becomes nuclear, we monitored the accumulation of survivin–GFP stably expressed in U2OS cells at intervals over 24 h (Fig. 1F,G). Maximum nuclear residence was achieved within 3 h and persisted until the end of the observational period (24 h). Interestingly, it also persisted after the cells were returned to a normoxic environment, which we followed with re-oxygenation for 6 h (Fig. 1H,I).

### Hypoxia upregulates expression of survivin and EZH2 and increases H3K27 trimethylation

**Fig. 2.**
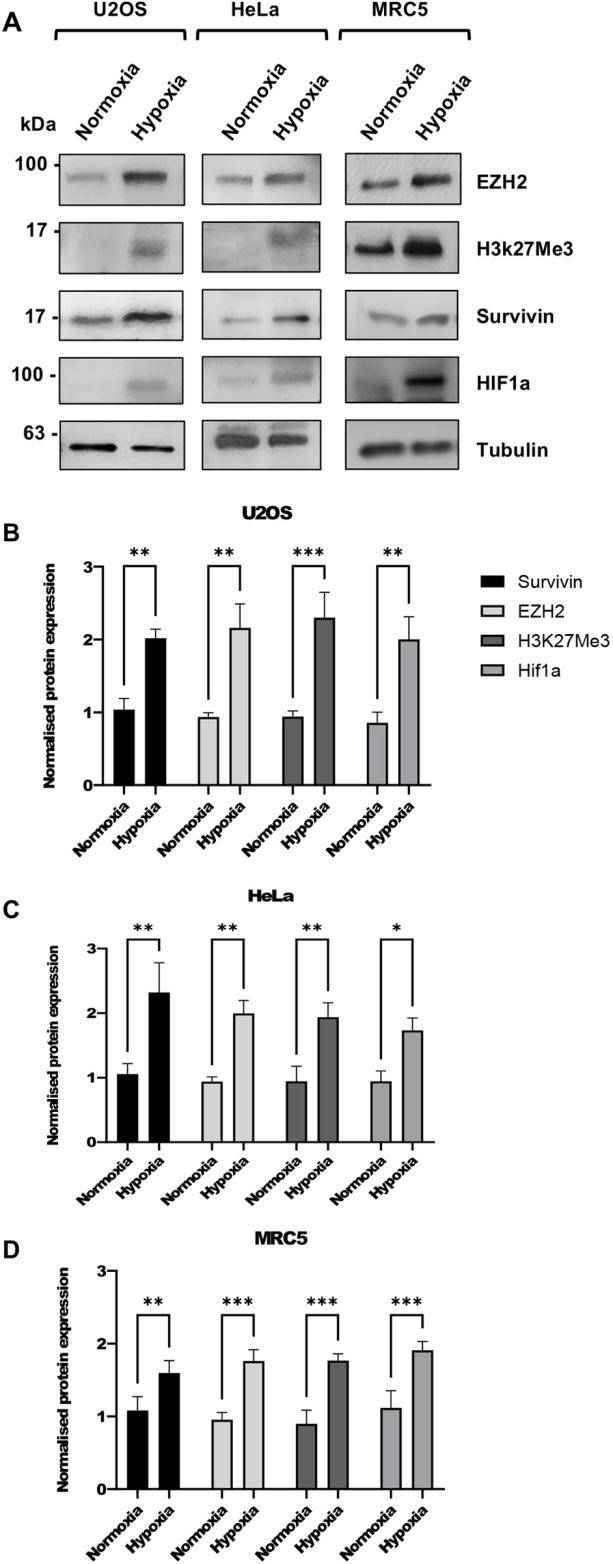
**Hypoxia increases expression of EZH2, H3K27me3 and survivin.** (A) Immunoblots of WCEs from U2OS, HeLa and MRC5 lines cultured under normoxic or hypoxic environments (24 h). Blots were immunoprobed with anti-EZH2, anti-H3K27me3 and anti-survivin antibodies. Anti-Hif1a used to prove the hypoxic state had been induced, and anti-tubulin was used as a loading control. (B–D) Quantification of immunoblots represented in A from three independent experiments demonstrating that EZH2, H3K27me3 and survivin are all more abundant under hypoxia. Data presented are means±s.d. *P<0.05, **P<0.01, ***P<0.001 (two-way ANOVA with Tukey's multiple comparisons post test).

Next, we investigated whether there was any correlation between survivin expression and the chromatin remodelling machinery. To do this we immunoblotted whole-cell extracts (WCEs) from three cells lines grown in normoxia or in hypoxia (24 h) with antibodies to survivin, EZH2 and H3K27me3 (Fig. 2). Using anti-HIF1a to ensure the hypoxic state had been induced and tubulin as a loading control, we found that expression of EZH2 and the abundance of H3K27me3 increased in all lines tested. Survivin expression also increased under hypoxia. These findings provided strong biological context for us to hypothesise that there might be cross talk between these proteins and histone tri-methylation.

### Survivin interacts with EZH2

**Fig. 3.**
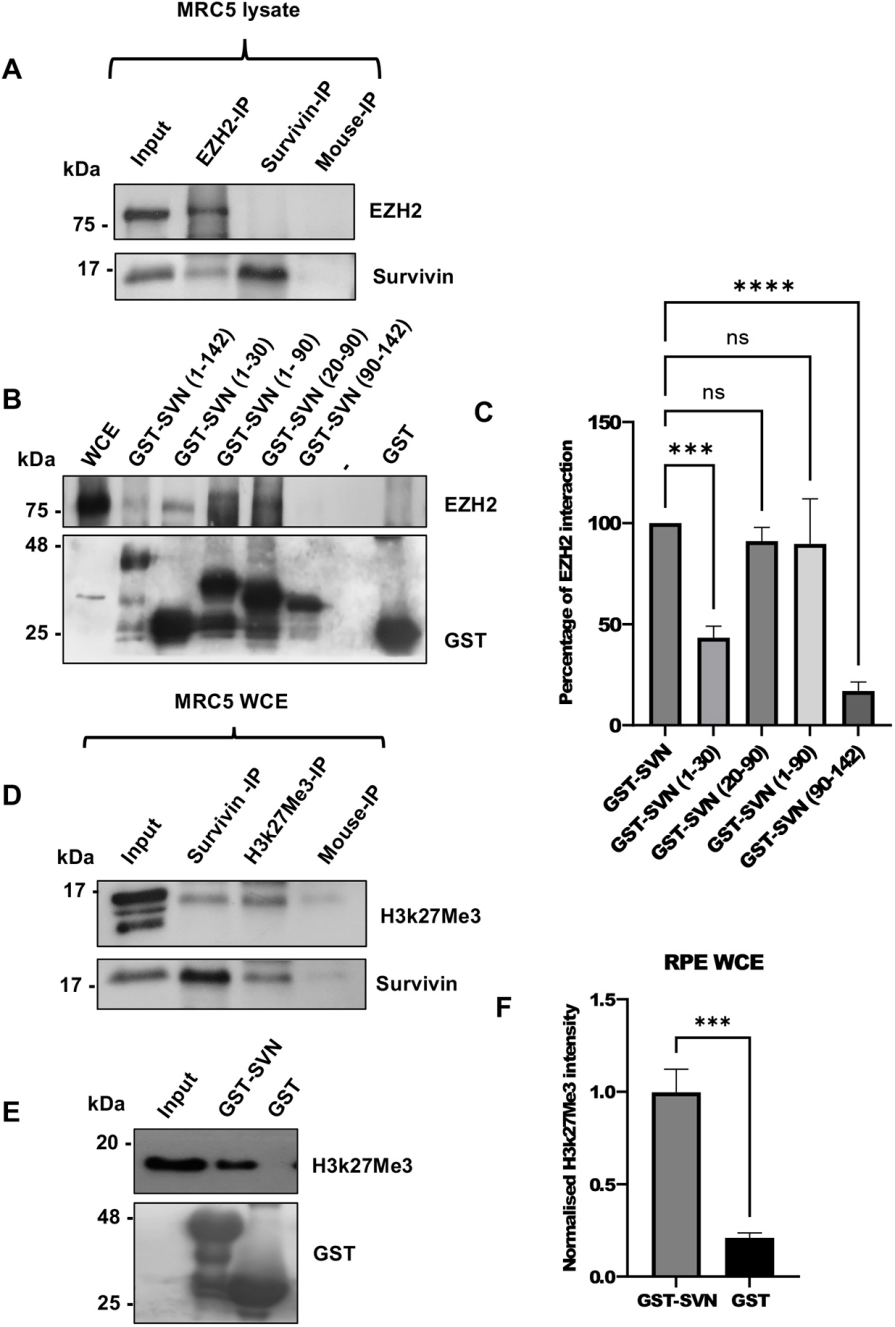
**Survivin and EZH2 interact.** (A) Immunoprecipitation was carried out using whole MRC5 extracts using anti-survivin (C60), anti-EZH2, mouse IgG antibodies (negative control). Co-immunoprecipitation was assessed with the alternative antibodies. Co-immunoprecipitation of EZH2 with survivin was evident when anti-EZH2 was used to immunoprecipitate but not when the anti-survivin (C60) antibody was used. (B) GST pulldown assay was carried out with WCEs prepared from RPE cells expressing GST (negative control), GST–survivin and various GST-tagged survivin truncations, (numbering indicating amino acids), used as bait. (C) Quantification of interactions represented in B. EZH2 binds mainly to the first 90 amino acids of survivin. Data are mean±s.d. from three independent experiments. ***P<0.001; ****P<0.0001; ns, not significant (one-way ANOVA with Dunnett's post hoc test). (D) Immunoprecipitation was carried out as in A but using anti-H3K27me3 specific antibodies, rather than anti-EZH2. Co-immunoprecipitation of survivin and H3K27me3 was evident in reciprocal samples. (E) The GST pulldown experiment as in B was repeated using RPE cell lysates with GST or GST–survivin, and interaction with H3K27me3 determined by immunoblotting. (F) Quantification of data represented in E, normalised to the GST or GST–survivin. Data are mean±s.d., *n*=3. ***P<0.001 (unpaired two-tailed Student's *t*-test). Blots in A and D are representative of three repeats. Inputs are 7.5%.

Having established a correlation in the expression of survivin, EZH2 and H3K27me3 under hypoxia, we next carried out a co-immunoprecipitation (co-IP) experiment with anti-EZH2 and survivin antibodies. As shown in Fig. 3A, anti-EZH2 successfully pulled down survivin in MRC5 lysates; however, C60-specific survivin antibody was unable to pulldown EZH2.

Next, we used a GST pulldown assay using recombinantly expressed GST and GST–survivin (GST–SVN) proteins and WCEs prepared from RPE (Fig. 3B,C; Fig. S2A,B), U2OS (Fig. S2C,D) and MCF7 cells (Fig. S2E,F). In each case a positive interaction between survivin and EZH2 was detected, indicating that this is a ubiquitous interaction, and not cell line specific. Subsequently we mapped the interaction domain(s) using the same assay and various truncated versions of survivin. As shown in Fig. 3B,C, amino acids 1–90 and just the BIR domain (20–90) had a strong affinity to EZH2, which was not significantly different to the interaction with the full-length protein. Quantification of these blots demonstrated that these domains were able to pulldown approximately 90% of EZH2 compared to full length survivin (Fig. 3C). As the anti-survivin antibody used in the immunoprecipitation in Fig. 3A was directed to the BIR domain (C60), this further supports the notion that the BIR domain is involved, as this would compete with EZH2 for interaction, hence our inability to detect the reciprocal co-IP.

### Survivin interacts with the transcriptional repression marker H3K27me3

We also investigated whether survivin could interact with the PRC2 product H3K27me3. Similar to the experiments described above for EZH2, we carried out reciprocal co-IPs (Fig. 3D) and a GST pulldown assays (Fig. 3E,F) and probed for H3K27me3. In these experiments extracts were prepared from MRC5 and RPE cells, and in each case interaction between survivin and H3K27me3 was observed.

### Reducing survivin expression increases tri-methylation of H3 at K27

The results thus far presented suggest that nuclear survivin has the potential to alter chromatin configuration. To address this, we measured the abundance of H3K27me3 in cell extracts before and after removal of survivin. First, we used the survivin suppressant drug sepantronium bromide (YM155), which reduces survivin transcription by binding to its promoter and abrogating Sp1 transcription factor activity (Cheng et al., 2012). U2OS and MRC5 cells were treated with YM155 for 48 h and immunoblotting confirmed that survivin levels decreased in a concentration-dependent manner (Fig. S3). However, a decrease in EZH2 protein was also detected in both cell lines, and although H3K27me3 levels were significantly reduced in U2OS cells with this treatment, the same was not true for MRC5 cells. As EZH2 was also affected, and YM155 is known to have off-target effects (Mazzio et al., 2018; Mondal et al., 2022; Rauch et al., 2014), we opted to use siRNA to knock down survivin instead.

**Fig. 4.**
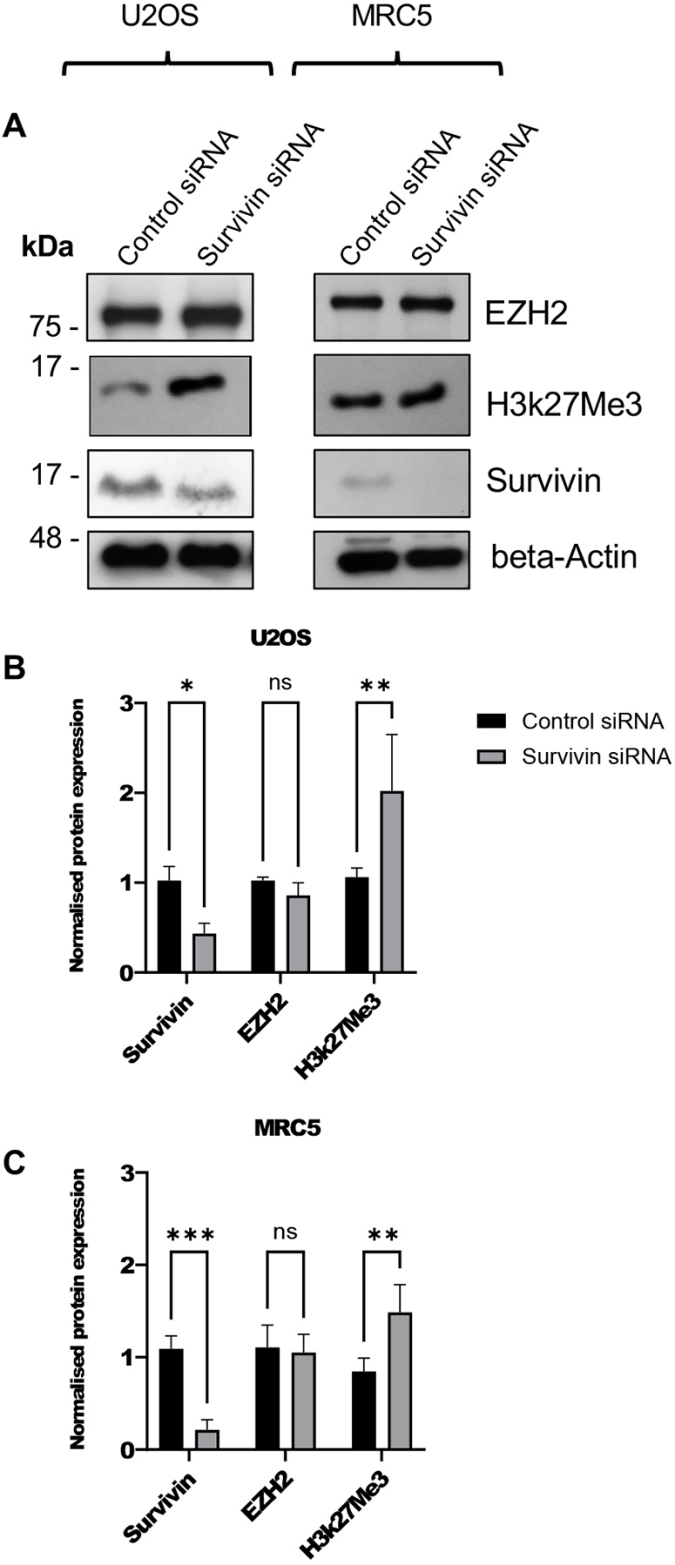
**Survivin knockdown increases H3K27me3 abundance.** (A) U2OS and MRC5 cells were incubated with control or survivin-specific siRNA for 48 h. Lysates were immunoblotted with antibodies against the indicated proteins. (B,C) Quantitative analysis of immunoblots, normalised to β-actin loading for (B) U2OS, and (C) MRC5 cells. No change was seen in EZH2 expression but H3K27me3 was increased in both lines. Data are means±s.d. from three independent experiments. *P<0.05; **P<0.01; ***P<0.001, ns, not significant (two-way ANOVA with Tukey's multiple comparisons post test).

Survivin-specific siRNAs were used as follows: silencer Select siRNA s1457 for U2OS cells and Silencer Select siRNA s1458 for MRC5 cells. As shown in Fig. 4, knockdown of survivin via siRNA was efficient in both lines, and unlike the YM155 treatment, it did not affect EZH2 protein levels. Notably, the abundance of H3K27me3 increased, suggesting that more inactive, possibly heterochromatic, DNA forms in the absence of survivin.

### mRNA transcripts of genes normally repressed by PRC2 are increased when survivin is knocked down

**Fig. 5.**
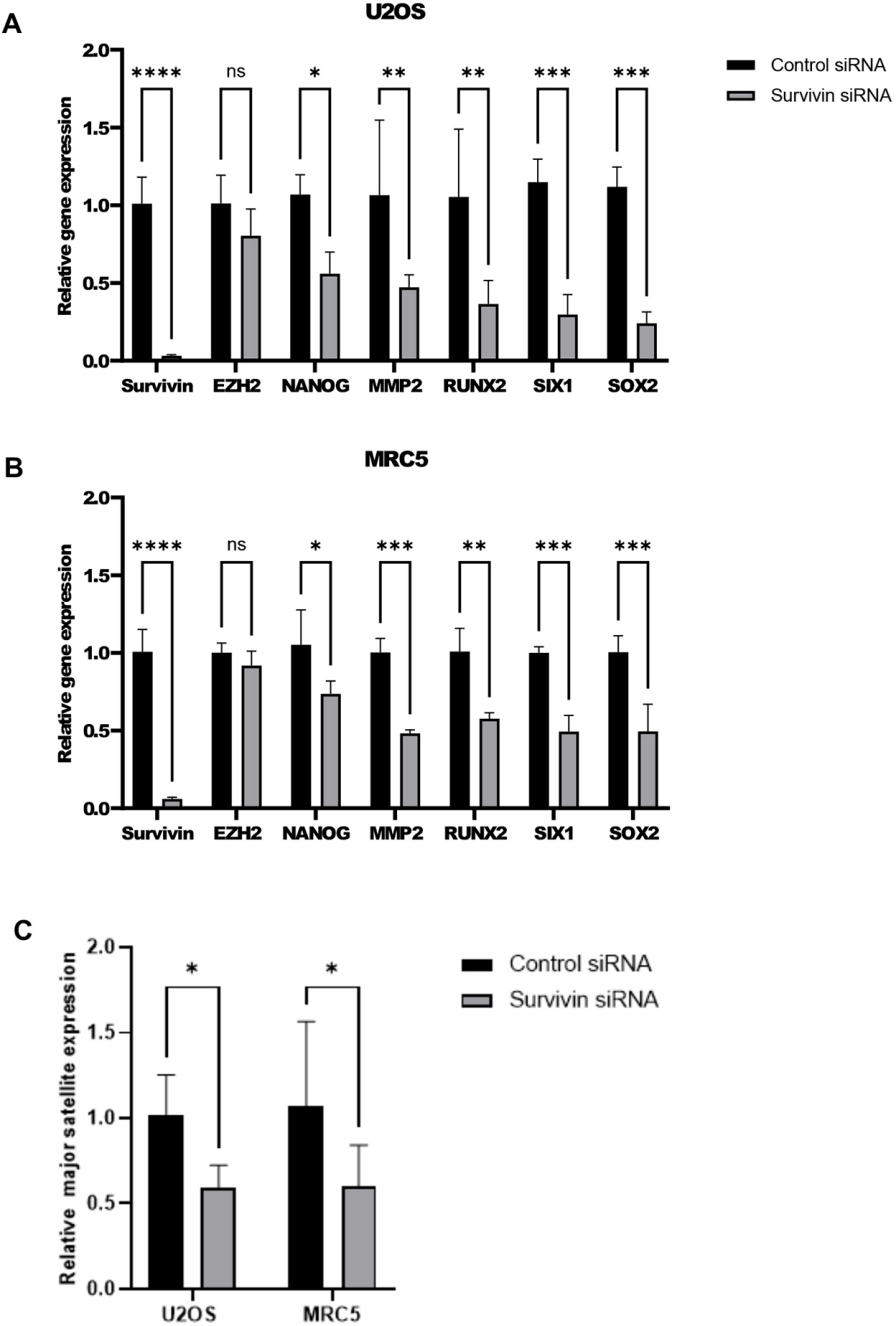
**Survivin knockdown reduces transcription of several key PRC2-dependent genes.** qPCR analysis was carried out for the genes indicated from (A) U2OS or (B) MRC5 cells treated with either control or survivin-specific siRNA (48 h). Data are normalized to control siRNA treatment and means±s.d. from *n*=3 plotted. *P<0.05; **P<0.01; ***P<0.001; ****P<0.0001; ns, not significant (two-way ANOVA with Tukey's multiple comparisons post test). (C) qPCR analysis of major satellite transcripts from U2OS or MRC5 cells exposed to control or survivin-specific siRNA. A significant reduction in major satellite expression occurred in the absence of survivin in both lines. Data are normalized to control siRNA treatment and means±s.d. from *n*=3 plotted. *P<0.05 (two-way ANOVA with Tukey's multiple comparisons post test).

As H3K27me3 levels increase following survivin knockdown, we next examined the impact of reduced survivin expression on PRC2 targeted genes using qPCR. Consistent with the immunoblotting, survivin transcripts were significantly reduced with the siRNA treatment, but EZH2 transcription was unaffected. Next, we analysed the expression of *NANOG*, *MMP2*, *RUNX2*, *SIX1* and *SOX2*, which all showed significantly reduced expression in the absence of survivin, in both cell lines (Fig. 5A,B).

### Major satellite mRNA expression is reduced when survivin is knocked down

Heterochromatin is recognised for its role in repressing pericentric major satellite transcription; accordingly, compromised heterochromatin is linked with increased transcription of major satellite (Maison and Almouzni, 2004; Mouse Genome Sequencing Consortium, 2002; Packiaraj and Thakur, 2024; Thakur et al., 2021; Baumann et al., 2023; Fuhrmann et al., 2025; Dutta et al., 2020). Thus, we next assessed major satellites mRNA expression by qPCR. As shown in Fig. 5C, knockdown of survivin reduced major satellite transcription, lending more weight to the notion that there is more inactive chromatin, possibly heterochromatin formed, and therefore more gene repression in the absence of survivin.

### Survivin association with EZH2 represses PRC2-mediated gene expression in pluripotent stem cells

**Fig. 6.**
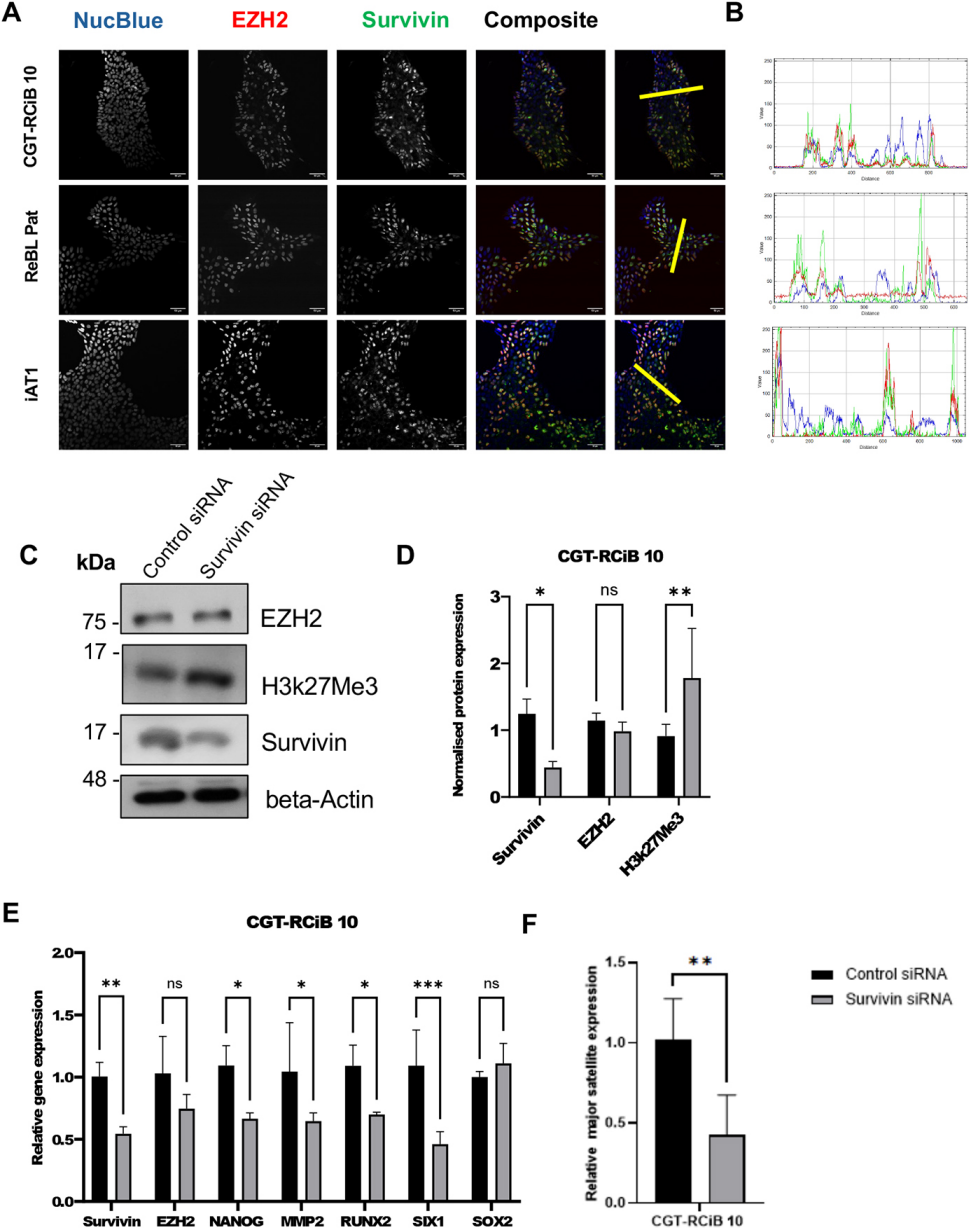
**Survivin and EZH2 in ihPSCs.** (A) Three pluripotent stem cell lines, CGT-RCIB 10, ReBL Pat and iAT1 were grown in normoxia and immunostained for EZH2 (red), endogenous survivin (green), and counterstained with NucBlue to show the nucleus (blue). Scale bars: 50 µm. (B) There is colocalisation of EZH2 and survivin in the nuclei as shown by the intensity profiles along the yellow line in A (FIJI software). Results representative of *N*=3 independent repeats. (C) CGT-RCIB 10 cells were incubated with control or survivin-specific siRNA for 24 h. Lysates were immunoblotted with antibodies against the indicated proteins. (D) Quantitative analysis of bands in immunoblots in C, normalised to the β-actin loading control. Although survivin was only partially knocked down, H3K27me3 abundance increased significantly. Data are normalized to control siRNA treatment and are means±s.d. from *n*=3 plotted. *P<0.05; **P<0.01; ns, not significant (two-way ANOVA with Tukey's multiple comparisons post test). (E) qPCR analysis was carried out for the genes indicated from CGT-RCIB 10 cells treated with either control or survivin-specific siRNA (24 h). Data are normalized to control siRNA treatment and means±s.d. from *N*=3 plotted. *P<0.05; **P<0.01; ***P<0.001; ns, not significant (two-way ANOVA with Tukey's multiple comparisons post test). (F) qPCR analysis of major satellite transcripts from CGT-RCIB 10 cells exposed to control or survivin-specific siRNA. A significant reduction in major satellite expression occurred in the absence of survivin. Data are normalized to control siRNA treatment and means±s.d. from *n*=3 plotted. **P<0.01 (unpaired two-tailed Student's *t*-test).

In addition to cancer cells and MRC5 cells, we looked at three human induced pluripotent stem cell (hiPSC) lines, CGT-RCIB 10, ReBL Pat and iAT1 cells, selected because they have high basal activity of PRC2 (Bernstein et al., 2006). Interestingly, immunofluorescence imaging revealed that survivin is expressed in the nucleus of all three stem cell lines grown in normoxia (Fig. 6A, green), where it colocalizes with EZH2, as demonstrated using a line profiling tool and overlapping peaks of red, green and blue signals (Fig. 6B). Next, we knocked down survivin by siRNA in the CGT-RCIB 10 line, using SignalSilence^®^ Survivin siRNA II. This 24 h treatment reduced but did not completely knock down survivin in these cells. Nevertheless, consistent with the other lines, immunoblotting showed no alteration in EZH2 expression and a significant increase in H3K27me3 after survivin depletion (Figs 6C,D). Moreover, qPCR analysis revealed reduced expression of *NANOG*, *MMP2*, *RUNX2* and *SIX1* by qPCR in the absence of survivin (Fig. 6E), although the effect was less pronounced than observed in MRC5 and U2OS cells and SOX2 was not affected. Finally, we looked at the expression of major satellite mRNA expression by qPCR in these pluripotent stem cells: despite the incomplete knockdown, we observed a significant reduction in expression of these transcripts (Fig. 6F). From these data we conclude that, survivin plays a role in PRC2-mediated gene expression in pluripotent stem cells.

## DISCUSSION

Transcriptional programming is regulated epigenetically at the chromatin level, often through post-translational modification of histones, the DNA-packing proteins that constitute the nucleosome. Genes in densely packed areas of chromatin, or ‘heterochromatin’, are generally repressed, and this density is due in part to modification of histones (Morrison and Thakur, 2021). The PRC2 complex tri-methylates H3 at lysine 27 causing the DNA to take on a closed conformation, or heterochromatic, configuration that represses many genes, some of which are involved in directing differentiation and/or tumour progression (Li, 2014; Laugesen et al., 2016; Piunti and Pasini, 2011; Comet et al., 2016). The differentiation genes that we have looked at here are *NANOG*, *RUNX2*, *SIX1* and *SOX2* (Zhang and Cui, 2014; Cavaleri and Scholer, 2003; Thomas et al., 2004; Le Grand et al., 2012; Liu et al., 2014). We also looked at *MMP2*, which encodes a metalloproteinase that degrades extracellular matrix and promotes cancer metastasis (Mustafa et al., 2022).

**Fig. 7.**
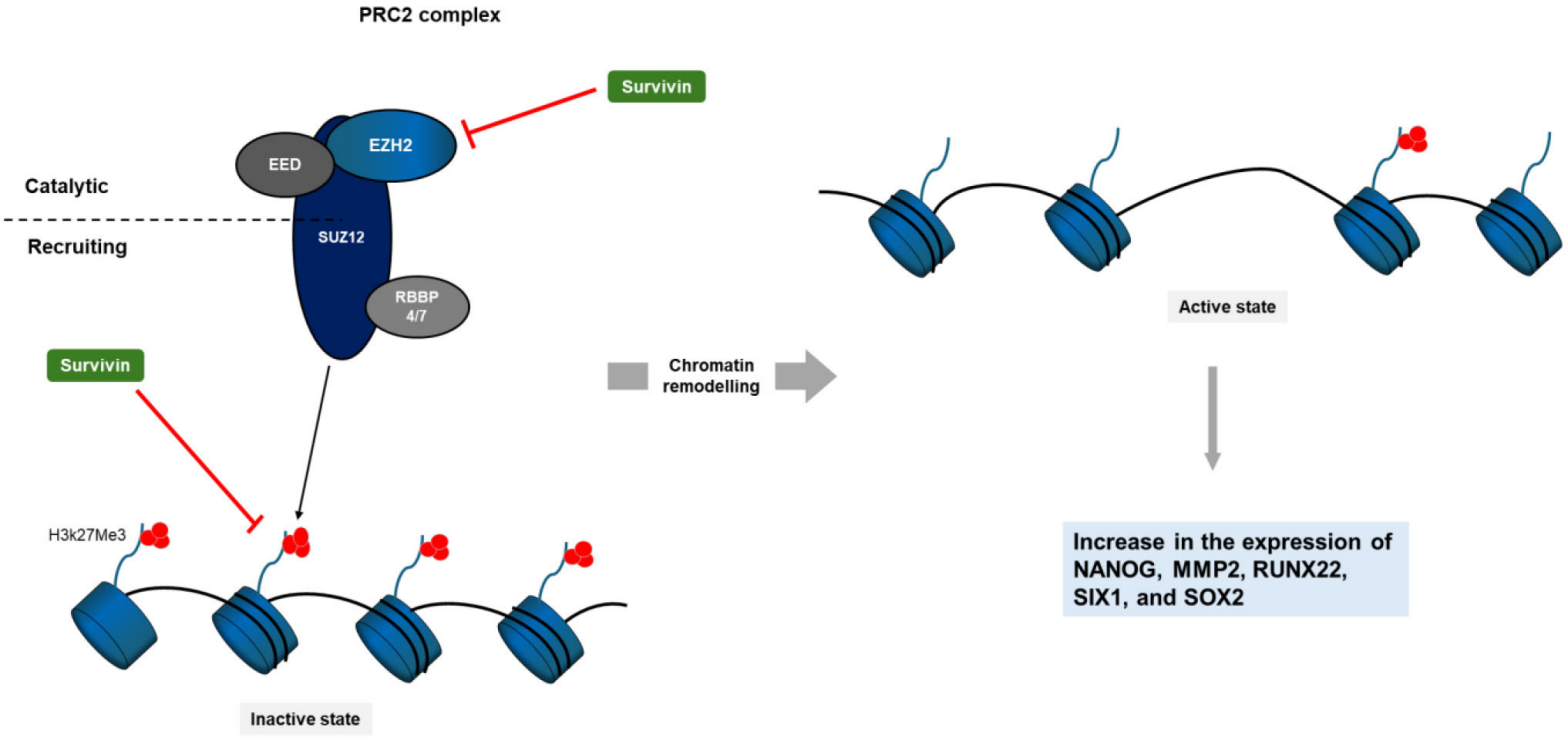
**Model of survivin–PRC2 interaction and its impact on transcription.** Survivin inhibits the PRC2 complex causing a reduction in H3K27 tri-methylation, which opens the DNA to enable transcription of genes that are normally repressed by PRC2.

In this study, we have reported a physical and functional relationship between survivin and EZH2, the catalytic subunit of PRC2, as well as H3K27me3 itself. To recap briefly, we showed that nuclear survivin can interact with EZH2, which we propose can cause a reduction in H3K27me3 and heterochromatin formation (Fig. 7). The interaction with tri-methylated H3 suggests a second way that survivin might interfere with this pathway and hold the chromatin in open configuration (Fig. 7). To our knowledge, this is the first study to demonstrate that survivin interacts with EZH2 and its histone product H3K27me3. Our results further show that when survivin is removed from a variety of cells, that more H3K27 trimethylation occurs, and therefore transcripts of pericentric major satellite and PRC2-regulated genes, listed above, are reduced. This novel mechanistic data firmly establishes survivin as a transcriptional co-regulator and supports recent genome-wide DNA-binding studies that showed survivin overlapping with repressed chromatin domains marked with H3K27me3 (Erlandsson et al., 2022).

Interestingly, H3K27me3 alterations are prevalent in paediatric brain tumours: 80% of diffuse midline gliomas and ependymomas harbour a methionine substitution for K27 (Wu et al., 2012; Argersinger et al., 2021). This K27M mutation causes a global loss of H3K27 trimethylation and this altered epigenetic profile can be used to distinguish them from other tumour types (Louis et al., 2021). Importantly, K27M holds the chromatin in an open configuration, leading to upregulation of many proto-oncogenes and downregulation of differentiation factors. In light of the data herein presented, it will be of great interest to determine how cells derived from these tumours respond to alterations in survivin expression, which might provide valuable insight that will enable us to ascertain whether the effects we have observed are due to its interaction with the PRC2 complex or the H3K27me3 mark itself.

We chose to work with hiPSCs as they have a high basal activity of PRC2, which represses transcription of genes associated with differentiation (Bernstein et al., 2006). These results suggest that in additional to its well-recognised involvement in cancer, survivin might play a more general role in regulating gene expression, and that this varies depending on the microenvironment, which could be of relevance during development and/or differentiation. Furthermore, the presence of survivin in nuclei of stem cells hints at a broader developmental or chromatin regulatory role, extending beyond its established functions in apoptosis and mitosis. Indeed, there are already some indications that survivin and PRC2 gene regulation is connected with ‘stemness’ – higher survivin levels have been associated with the maintenance of pluripotency in hiPSCs (Gil-Kulik et al., 2019), and its expression is ninefold higher human embryonic stem cells than in differentiated cells (Mull et al., 2014). Survivin is also implicated in cancer stem cell (CSC) biology, a subpopulation of tumour cells with self-renewal capabilities and resistance to apoptosis, behaviours linked to cancer reemergence (Mertins, 2014). In accord with this study, changes in RUNX2 (Liao et al., 2010), SOX2 (Luo et al., 2019) and NANOG (Mull et al., 2014) expression have been reported to be influenced by high levels of survivin in CSCs. Thus, beyond the findings herein presented, our study suggests a role of survivin in chromatin remodelling more generally and linked to stemness.

To conclude, we show for the first time that survivin interacts with EZH2 and H3K27me3. Our data suggest that expression of survivin promotes the open chromatin state as depletion of survivin increases the incidence of closed chromatin or heterochromatin, and PRC2-mediated gene repression. This novel epigenetic role positions survivin as a dual regulator of genome stability and transcriptional plasticity, with implications for both normal development and cancer progression.

## MATERIALS AND METHODS

### Cell culture and drug treatment

Human cervical cancer cells (HeLa), human bone osteosarcoma cells (U2OS), retinal pigment epithelial cells (RPE) and human breast cancer cells (MCF7) were originally from ATCC. Human embryonic lung fibroblasts (MRC5) were sourced from the Genome Damage and Stability Centre, Sussex, UK. These cells were cultured in Dulbecco's modified Eagle's medium (DMEM; Sigma-Aldrich, D6429) containing 4500 mg/l glucose, sodium pyruvate, L-glutamine and sodium carbonate (Sigma, D6429), supplemented with 10% fetal bovine serum (Sigma, F7524) and antibiotic-antimycotic solution containing 10,000 units/ml penicillin, 10 mg/ml streptomycin and 25μg/ml Amphotericin B per ml (Sigma, A5955) (referred to as complete medium). For standard culture and experimentation, cells were cultured at 37°C in a 5% CO_2_ (20.6% O_2_) in a humified environment, hereafter referred to as ‘normoxia’. For hypoxic experiments, cells were cultured in hypoxic chamber (Mark Coy Lab Products) at 37°C in a 5% CO_2_ humified environment with 1% O_2_, hereafter referred to as ‘hypoxia’. hiPSCs were CGT-RCIB 10, ReBL Pat and iAT1 cells (gifts from Prof. C. Denning, University of Nottingham, UK) were maintained in Essential 8™ (E8) medium (Thermo Fisher Scientific #A1517001) in Matrigel™-coated (1:100 dilution, BD Biosciences #356235) cell culture dishes. Derivative U2OS cell lines expressing GFP or survivin–GFP constructs were cultured under selective pressure of 1 mM G418 (ACROS organics) in complete medium. Cells were passaged at 70–80% confluency, washed with Dulbecco's phosphate-buffered saline (DPBS; Sigma, D8537), and harvested using trypsin-EDTA solution (Sigma, T4174) before being seeded into culture plates (Thermo Fisher Scientific). Mycoplasma contamination testing was conducted on all cells every three months.

YM155 (Selleck Chemicals) was solubilised in DMSO (Sigma) and used at 0–100 nM as indicated over 24 or 48 h.

### Microscopy techniques

#### Sample preparation

Half a million cells were seeded onto sterile poly-lysine coated coverslips in 12-well plates and incubated overnight. Cells were washed with pre-warmed PBS then fixed in 4% formaldehyde solution [10 min, room temperature (RT)], followed by 3×5-min PBS washes. Cells were then permeabilised with PBS/0.15% Triton X-100 (Sigma, T8787) for 10 min (RT), and blocked for 30 min in PBA buffer [PBS with 1% BSA (Merck) (w/v) and 0.1% Azide (Merck) (v/v)], at RT. Next, they were incubated with primary antibody diluted in PBA buffer (1 h, RT). After 3 more washes in PBS they were incubated (1 h, RT) with the appropriate fluorescently labelled-secondary antibodies diluted in PBA. Samples were counterstained in 1 µg/ml DAPI or NucBlue (Thermo Fisher Scientific) in PBS (10 min, RT) to stain nuclei, washed again in PBS then mounted using Mowiol mounting medium (Sigma-Aldrich, 81381). The following primary antibodies were used at 1:200: anti-survivin (Cell Signaling Technologies 71G4B7 or 6E4), anti-H3K27Me3 (Abcam, Ab192985), anti-EZH2 [Cell Signalling Technologies (CST), D269]. Secondary antibodies diluted 1:1000 as appropriate were: anti-rabbit-IgG or anti-mouse-IgG conjugated to fluorescein or Texas Red (Vector Labs).

Images were acquired on a Zeiss 200 M microscope using a 20× (air 0.5 NA) or 40× (oil 1.3 NA) objective. The signal intensity of the secondary fluorescent antibodies or fluorophore-only controls was used to determine the minimum signal threshold for the channels. The DAPI- or NucBlue-positive region was used to evaluate survivin intensity within the nucleus through colocalization analysis of the blue and green channels, respectively. The Pearson correlation coefficient was obtained using the Fiji plugin called ‘Just Another Colocalisation Plugin’ (BiOP-JACoP). An intensity plot profile was generated using the Fiji software for the region of interest within the cell. All measurements were collected in Microsoft Excel and transferred to GraphPad Prism software for analysis.

### Preparation of protein cell extracts and subcellular fractionation

For the preparation of whole-cell extracts (WCEs), cells were cultured to ∼80% confluency, harvested by scraping into ice-cold PBS solution, pelleted (1000 ***g*** for 5 min at 4°C) then resuspended and lysed on ice (30 min) in RIPA buffer (10 mM Tris-HCl pH 7.5, 0.5 mM EDTA, 150 mM NaCl, 0.1% SDS, 1% sodium deoxycholate and 1% Triton X-100) supplemented with a protease inhibitor cocktail [2 mM β-glycerophosphate, 1 µg/ml 1:1:1:1 chymostatin:leupeptin:antipain:pepstatin A (CLAP), 100 µm phenylmethylsulphonyl fluoride (PMSF), 20 mM Na_3_VO_4_, 2 mM MgCl_2_ and 4 U/ml DNase (Quanta Biosciences)]. After lysis samples were sonicated at 50 Hz (10 s) and 25 Hz (10 s) on ice. Protein concentration was determined with a Bradford assay. Cell lysates intended for pulldown assays were stored at −20°C, whereas lysates for SDS-PAGE were boiled (95°C, 5 min) with loading buffer (SDS sample buffer) [100 mM β-mercaptoethanol, 50 mM Tris-HCl pH 6.8, 10% glycerol (w/v), 2% SDS (w/v) and 0.01% Bromophenol Blue (w/v)].

For subcellular fractionation, cells were harvested and centrifuged at 1000 ***g*** for 5 min. The extraction buffer [1.5 mM mannitol, 0.5 mM sucrose, 10 mM ethylene glycol-bis(b-aminoethyl ether)- N,N,N′,N′-tetraacetic acid, 10 mM 4-(2-hydroxythyl)-1-piperanzineethanesulfonic acid (HEPES), pH 7.5] was supplemented with an inhibitor cocktail (as above for RIPA buffer). This buffer was used to resuspend and lyse the sample in a glass homogeniser (Teflon). A small aliquot of the lysed samples was collected as the WCE, whereas the remainder was centrifuged at 1000 ***g*** (5 min, 4°C). The supernatant containing the cytoplasmic fraction, was collected in a separate tube, and the pelleted nuclear fraction was washed three times with extraction buffer. Furthermore, the nuclear fraction lysates were subjected to at 50 Hz sonication for 3×10 s to shear DNA (Vibra-Cell™ Ultrasonic), followed by centrifugation at 18,000 ***g*** (15 min, 4°C), after which supernatant was collected. The cytoplasmic supernatant was centrifuged at 1000 ***g*** (5 min, 4°C) and supernatant was collected in separate tube to remove nuclear contamination. The final protein concentration was determined by Bradford assay, and the samples were reduced in SDS sample buffer as for WCEs.

### Immunoblotting

To separate proteins, 20 µg of protein was loaded per lane onto 12% or 15% SDS-PAGE gels and electrophoresed in running buffer [192 mM glycine (Sigma, G8898), 25 mM Trizma, 3.5 mM SDS (Sigma, 74746)], at 120 V for 20 min, followed by 200 V for 40 min. Proteins were transferred onto 0.22 µm nitrocellulose membrane (Amersham Biosciences) at 350 mA (1.5 h) in transfer buffer [192 mM glycine, 25 mM Trizma, 10% methanol (v/v)]. The membranes were subsequently blocked in suitable blocking solution: 5% non-dry milk (Marvel) or 5% BSA (Sigma, A7906) in Tris-buffered saline with Tween-20 (Sigma, P1379) [TBST; 150 mM NaCl, 50 mM Trizma, 5% non-fat dried milk (w/v) or 5% BSA (w/v), 0.1% Tween-20 (v/v) pH 7.4 with HCl] for 1 h with shaking. After blocking, membranes were rinsed with TBST and incubated with appropriate primary antibody at 4°C overnight. Primary antibodies were diluted 1:1000 in TBST with 5% milk, unless otherwise stated, and were against: tubulin (Sigma, B512, T5168), β-actin (Invitrogen MA1-140), TBP (CST, 8515), survivin (C60, CST 71G4B7, TBST 2% milk; or 6E4), H3K27me3 (Abcam, ab192985; TBST-2% BSA), GST (Cytivia, RPN1236V), EZH2 (CST, D269 or Proteintech 21800-1-AP), Hif1α (Novus Biologics, NB100-449). The following day, membranes were washed three times with TBST (5 min, RT), followed by incubation with HRP-conjugated secondary antibodies for 1 h at RT. Secondary antibodies (anti-mouse-IgG and anti-rabbit-IgG; DAKO, P0260 and P0217, respectively) were diluted 1:1000 in TBST with 5% milk. The membranes were washed again three times with TBST (5 min, RT) then incubated in the dark for 5 min with 1:1 mix of ECL reagents (Amersham Biosciences, RPN 2106). Finally, the membranes were exposed to Hyperfilm (Amersham Biosciences) in the dark for 2, 5, 10 and 20 min. The films were developed using developer and fixer solution (Harman). Protein bands were analysed using the Fiji software. Protein quantification was measured and normalised against the GST bands in the GST pulldown assays or against the loading controls (tubulin or actin) in the WCE blots. Uncropped images of blots from this work are shown in Fig. S4.

### Co-immunoprecipitation

Protein A/G agarose beads (Pierce, Thermo Fisher Scientific) were washed once with sterile H_2_O and twice with cold PBS. The lysates were cleared following 1 h incubation with the pre-washed beads, followed by centrifugation for 300 ***g*** at 4°C for 5 min. The cleared lysates (200 µl) were then incubated overnight with respective antibodies at 4°C on a rotor. The following day, the beads were washed as mentioned above and incubated with the overnight lysate (immunocomplex) for 2 h (RT) on a shaker. The beads were washed 3× with cold PBS, then pelleted through centrifugation at 300 ***g*** (4°C, 3 min). Proteins were subsequently eluted using 2× SDS sample buffer at 95°C for 5 min. All antibodies were used according to the manufacturer's IP protocol [against H3K27me3 (Abcam, ab192985), EZH2 (Cell Signaling Technologies, D269) and mouse IgG (Proteintech. 66360-1-IgG)].

### Recombinant protein expression

Standard heat-shock was used to introduce the relevant plasmid constructs into BL21 (DE3 *E. coli*) pLysS cells, these were pGEX4T1 (GST, Pharmacia), and pGEX4T1 with the following survivin variants GST–SVN, GST–SVN1-10 (Dunajová et al., 2025), GST–SVN1-30, GST–SVN20-90 (Abdelkabir et al., 2025), GST–SVN1-90 and GST–SVN90-142 (Abdelkabir et al., 2025; Dunajová et al., 2025). Transformed *E. coli* were plated onto LB agar plates supplemented with 50 µg/ml ampicillin. A single colony was used to inoculate larger cultures at 37°C until an optical density at 600 nm (OD_600_) of 0.6 was achieved. Protein expression was induced using 0.5 mM IPTG, and cultures incubated overnight at 20°C with shaking at 220 rpm. The bacterial cells were pelleted by centrifugation at 1000 ***g*** for 20 min at 4°C, washed with ice-cold TBS (20 mM Tris-HCl, pH 8.0, 150 mM NaCl) and resuspended in 20 ml of ice-cold TBS containing 1 µg/ml CLAP (chymostain, leupeptin, antipain, pepstain A), 4 U/ml deoxyribonuclease (DNase; Sigma), 2 mM MgCl_2_ (BDH) and 1 mM β-mercaptoethanol. Bacterial lysis was performed using sonication (Vibra-Cell™ Ultrasonic) for ten cycles of 10 s each at 25 Hz on ice, with 1 min intervals. The lysed cells were centrifuged twice at 4800 ***g*** for 30 min, and the supernatant was incubated with glutathione–Sepharose 4B beads (Cytiva, 17075601) for 1 h on a rotary wheel at 4°C. The beads, which bound GST-tagged proteins, were washed with TBST and TBS overnight and subsequently collected in elution buffer (10 mM glutathione, 50 mM Tris-HCl pH 8). Glutathione was removed from by dialysis using cellulose tubing (flat, width 10 mm, Sigma) in 2 l of dialysis buffer containing 50 mM Tris-HCl pH 7.4 and 150 mM NaCl, overnight with stirring at 4°C. The concentration of purified proteins was determined using the Bradford assay.

### GST pulldown assay

Glutathione–Sepharose 4B beads (100 µl) were washed with TBS, then centrifuged at 500 ***g*** (5 min, 4°C). WCEs were precleared with washed beads on a rotor at 4°C. For every 100 µl of GST beads, 500 µg of GST purified proteins were added and incubated with rotation for 1 h (4°C). After incubation, beads were washed once with TBST and twice with TBS, and centrifuged then washed with RIPA washes: RIPA wash buffer 1 [50 mM Tris-HCl, pH8.0, 150 mM NaCl, and 1% NP-40] and RIPA wash buffer 2 [50 mM Tris-HCl, pH8.0, and 1% NP-40]. Bound proteins were eluted from beads by boiling them with 100 µl of 2× SDS sample buffer (95°C, 5 min).

### siRNA transfection

Cells were cultured to 60–70% confluency, then transfected with siRNA using Lipofectamine RNAiMAX transfection reagent (Thermo Fisher Scientific). The siRNA survivin (s1457 and s1458 from Thermo Fisher Scientific), SignalSilence^®^ Survivin siRNA II (Cell Signalling Technology) and siRNA control (Thermo Fisher Scientific) were diluted in medium without antibiotics and supplemented with RNAiMAX transfection reagent for 10 min. This mixture was then added dropwise to the cells in antibiotic-free medium and cells analysed 24 or 48 h later.

### qPCR

#### Template preparation

Cells were washed twice with diethyl pyrocarbonate (DIPC; Sigma D5758) treated PBS, then harvested in 1 ml of DIPC-treated PBS and centrifuged at 1000 ***g*** for 5 min at 4°C. They were then lysed, and RNA extracted using a QIAGEN RNA extraction kit (RNeasy^®^ Micro kit, 74004) in accordance with the manufacturer's instructions. The samples were treated with DNase I, using the RNase free DNase kit (Qiagen1023460) and RNA concentration determined with a Nanodrop 2000 (Thermo Fisher Scientific).

Reverse transcription of RNA was performed using PrimeScript™ RT Master Mix kit for cDNA synthesis (TaKaRa, RR036A). A total of 500 ng of RNA samples were run in duplicate with one reaction containing PrimeScript™ RT Master Mix (RT+) and second lacking PrimeScript™ RT Master Mix (RT−). The samples were subjected to thermal cycling using a Bio-Rad thermocycler.

#### Primer design and efficiency

The gene Reference Sequence was identified using the Ensemble database and subsequently used in NCBI Primer-BLAST (Ye et al., 2012) with modified default settings including an optimal melting temperature (Tm) of 60°C, maximum PCR product size of 250 bp, and positioning of primers on exon-exon junctions. Primers to amplify specified genes were as follows: EZH2: fwd 5′-TGCTTCCTACATCGTAAGTGC-3′, rev 5′-CCTTTGCTCCCTCCAAATGC-3′; Tubulin: fwd 5′ ACTTTGTATTTGGTCAGTCT-3′, rev 5′-TTGCTGATAAGGAGAGTGCCC-3′; Actin: fwd 5′-GCAGAAAACAAGATGAGATTGGC-3′, rev 5′-TGTGAACTTTGGGGGATGCT-3′; MMP2: fwd 5′-CTGAGGGCGCTCTGTCTC-3′, rev 5′-TAGAAGGTGTTCAGGTATTGCACTG-3′; BIRC5 (survivin) fwd 5′-AGGACCACCGCATCTCTACA-3′; rev 5′-TATGTTCCTCTATGGGGTCGT 3′; GAPDH: fwd 5′-CAACAGCCTCAAGATCATCAGC 3′, rev 5′-TGGCATGGACTGTGGTCATGAG-3′; SIX1: fwd 5′-CAAGAACGAGAGCGTACTCAAGGC-3′, rev 5′-GGTGGTTGTGAGGCGAGAACTG 3′; SOX2: fwd 5′ CAGCATGTCCTACTCGCAGCAG-3′, rev 5′-CTGGAGTGGGAGGAAGAGGTAACC 3′; NANOG: fwd 5′ TATGCCTGTGATTTGTGGGC-3′, rev 5′-GGTTGTTTGCCTTTGGGACT-3′; and major satellite: fwd 5′-AGGGAATGTCTTCCCATAAAAACT-3′, rev 5′-GTCTACCTTTTATTTCAATTCCCG-3′.

The efficiency of the primers was assessed by quantitative analysis of the threshold breaking cycle (Cq) for 6 cDNA templates obtained from 1:2 serial dilutions. The reaction utilised 1.25 ng/µl of forward and reverse primers, with iTaq Universal SYBR Green Supermix. The samples were run in duplicates alongside negative controls: RT- and RNase-free water, using a thermocycler (QuantStudio 5, Thermo Fisher Scientific). The Cq values were quantitatively analysed, and primer efficiency was determined using QuantStudio 5 software.

qPCR was performed using the iTaq Universal SYBR Green Supermix (Bio-Rad) according to the manufacturer's guidelines. The cDNA was used at 0.5 ng/µl, and the samples were processes in QuantStudio 5 thermocycler (Thermo Fisher Scientific). Data analysis was conducted as outlined in references (Vandesompele et al., 2002; Hellemans et al., 2007).

### Statistical analyses

All statistical analyses were performed using GraphPad Prism 10. Data are presented as mean±s.d. from at least three independent experiments (unless stated otherwise in the respective figure legends). The normality of the data and equality of variances were evaluated using the Shapiro–Wilk and *F*-test, respectively. Comparisons between two sets were analysed using the Student's unpaired two-tailed *t*-test. One-way ANOVA was used for data sets containing more than two samples. For more complicated methodologies involving repeated measurements, two-way ANOVA was conducted. ANOVA was followed by either post hoc Tukey's multiple comparisons test or Dunnett's multiple comparisons test, contingent upon whether all samples were compared against each other or against a control sample, respectively. *P*<0.05 was considered statistically significant. Exact *P*-values and sample sizes (*n*) are reported in the figure legends.

## Supplementary Material

10.1242/joces.264572_sup1 Supplementary information
