# Supplementary material for: Interplay between nuclear survivin and the PRC2 complex and its impact on H3K27me3-directed transcriptional repression
Source: J Cell Sci. 2026 Mar 17;139(12):jcs264572. doi: 10.1242/jcs.264572 (PMC13035274; doi:10.1242/jcs.264572)
Supplement: Supplementary information [file joces-139-264572-s1.pdf]

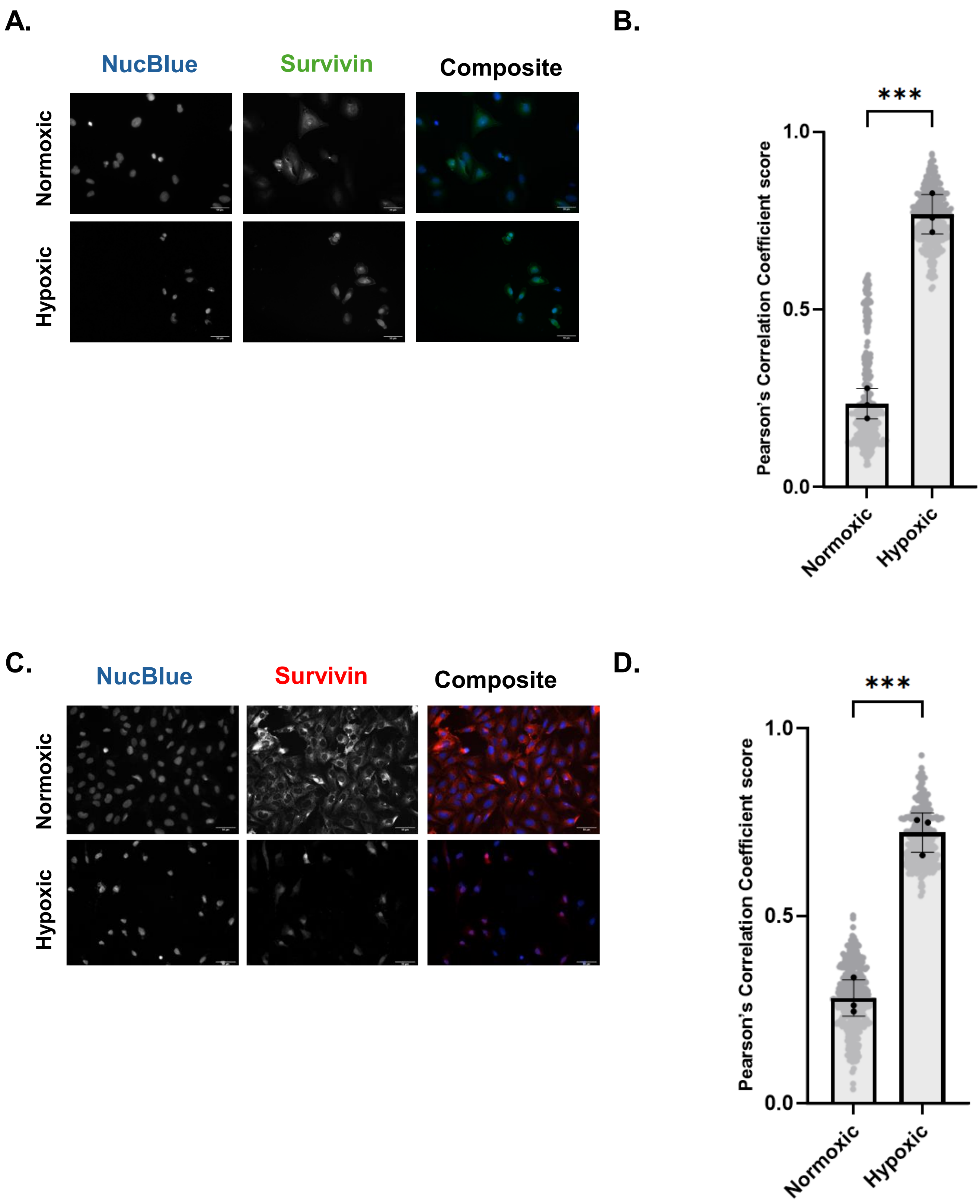

Fig. S1. Endogenous survivin is nuclear in HeLa cells under hypoxia. (A) HeLa cells were subjected to hypoxia for 24h then fixed and immunostained for endogenous survivin (red) and counterstained with NucBlue (blue) to show the nuclei. Images are representative of three independent experiments (scale bar = 50 µm). (B) Co-localisation analysis of images represented in (A) was carried out using Pearson's correlation coefficient scores. Statistical analysis was performed using an unpaired Student's t-test. (C and D). MRC5 cells treated as above, but a red secondary antibody was used. Survivin was significantly more nuclear when cells were exposed to hypoxia. (Data are mean +/- SD, ****P < 0.0001, n=250 cells).

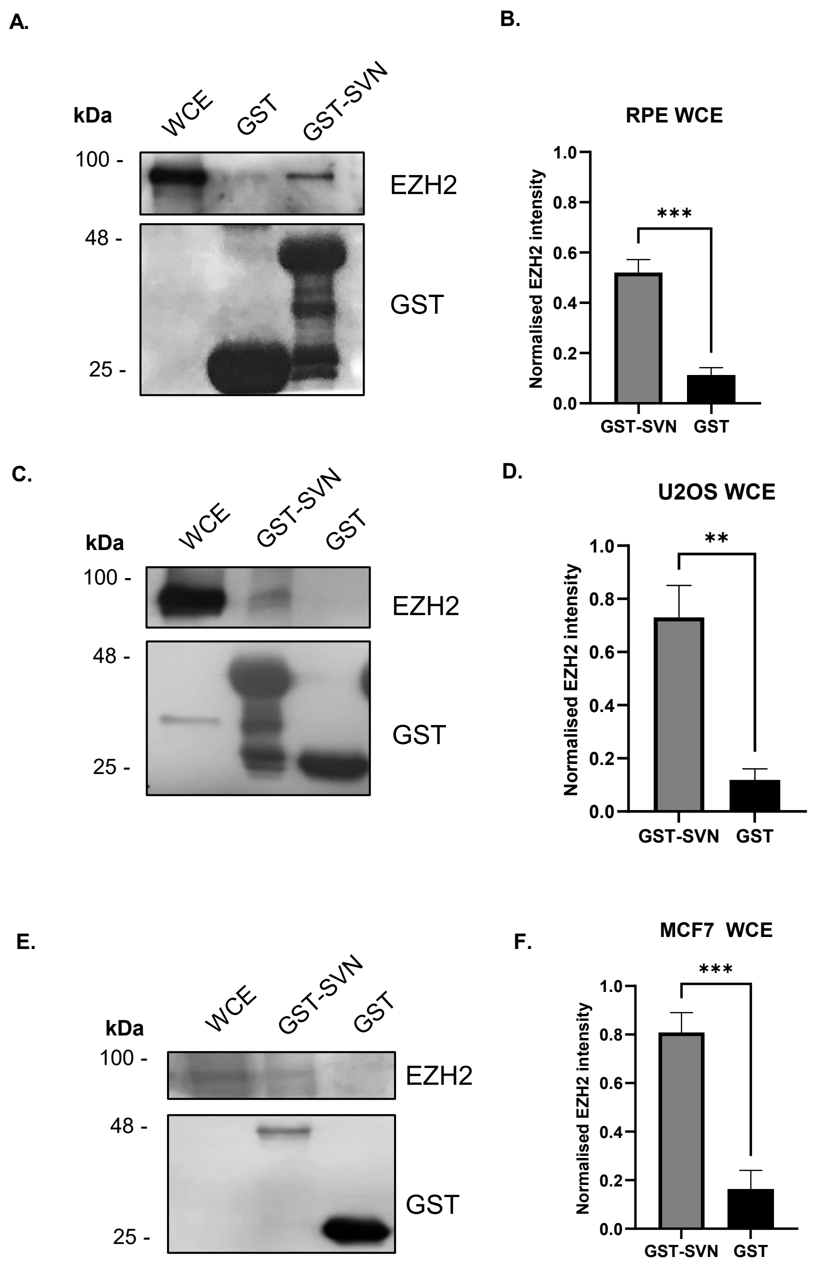

Fig. S2. Survivin interacts with EZH2 in normal and cancerous cell lines. GST pull down with GST alone (negative control), and full-length GST-survivin, incubated with extracts from (A&B) RPE, (C&D) U2OS, and (E&F) MCF7 cells. A significant interaction was observed in each line. Statistical analysis was performed using an unpaired Student's t-test . Data in graphs are normalised to GST/GST-survivin bands and means +/- SD of N=3 independent experiments shown (**p<0.01, ***p<0.001).

Journal of Cell Science • Supplementary information

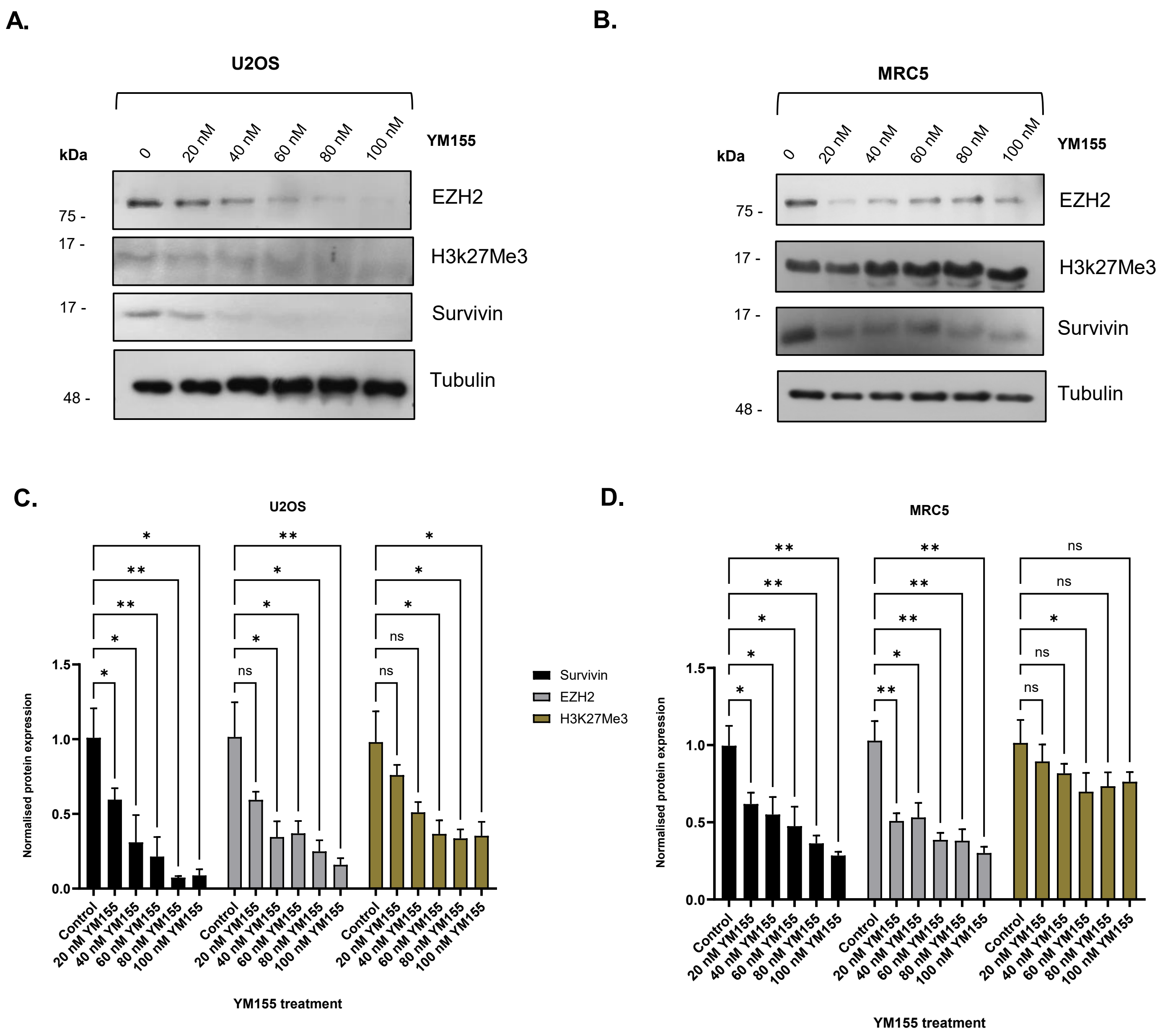

Fig. S3. YM155 reduces survivin, EZH2 and H3k27Me3 expression. Immunoblot analysis of EZH2, H3K27Me3 and survivin expression in U2OS cells **(A)** and MRC5 cells **(B)** treated with varying concentrations of YM155 for 48h. **C & D.** Quantitative analysis of blots represented in **(A&B)**, normalised to the tubulin loading control. Data are means ± SD, from N=3 (*p<0.05, **p<0.01, ***p<0.001, ****p<0.0001; ns = not significant).

C.

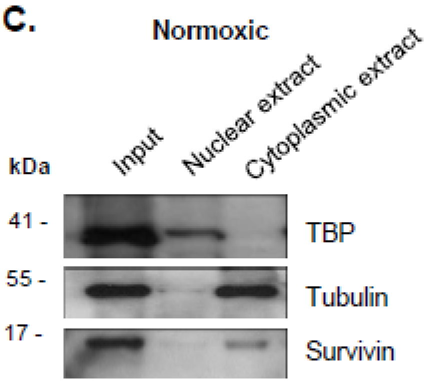

D.

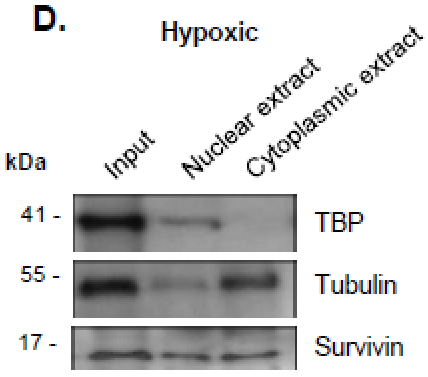

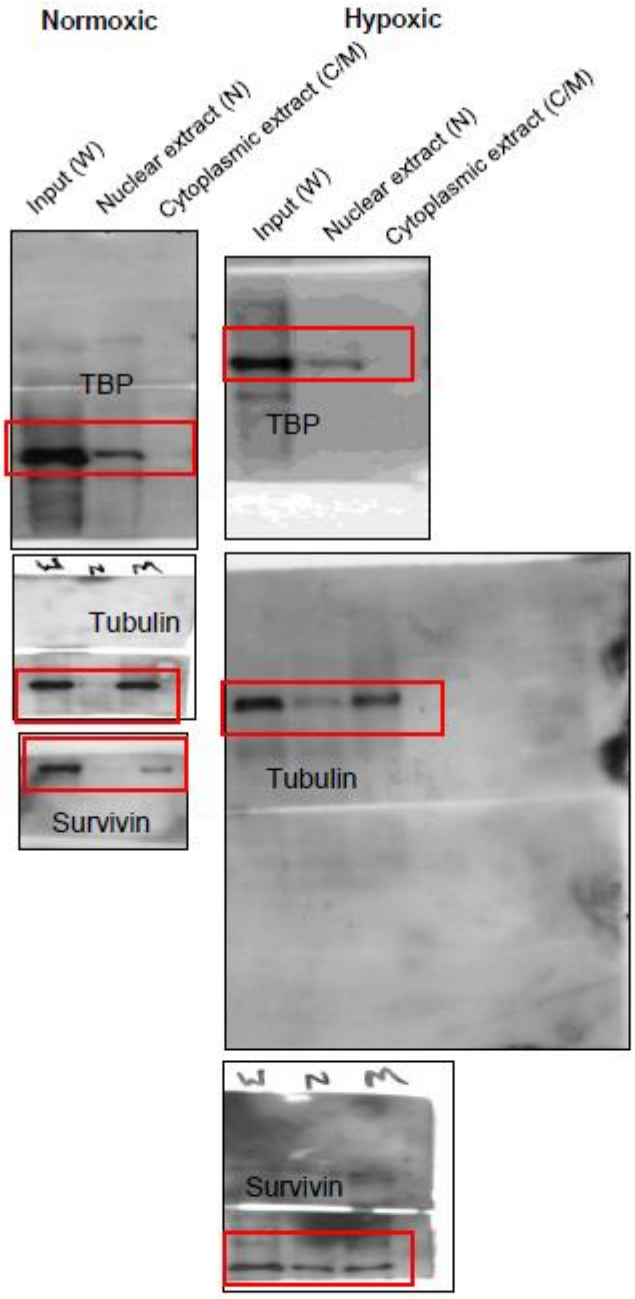

Journal of Cell Science • Supplementary information

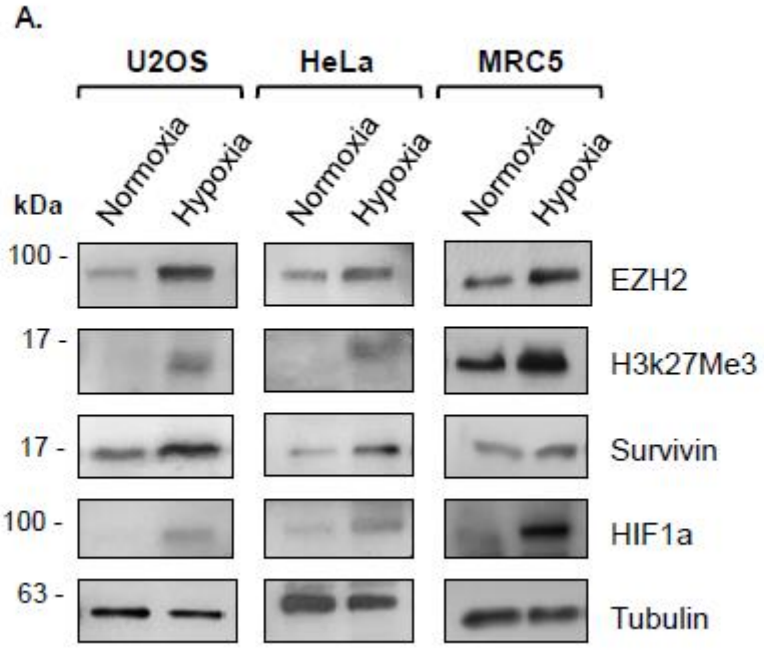

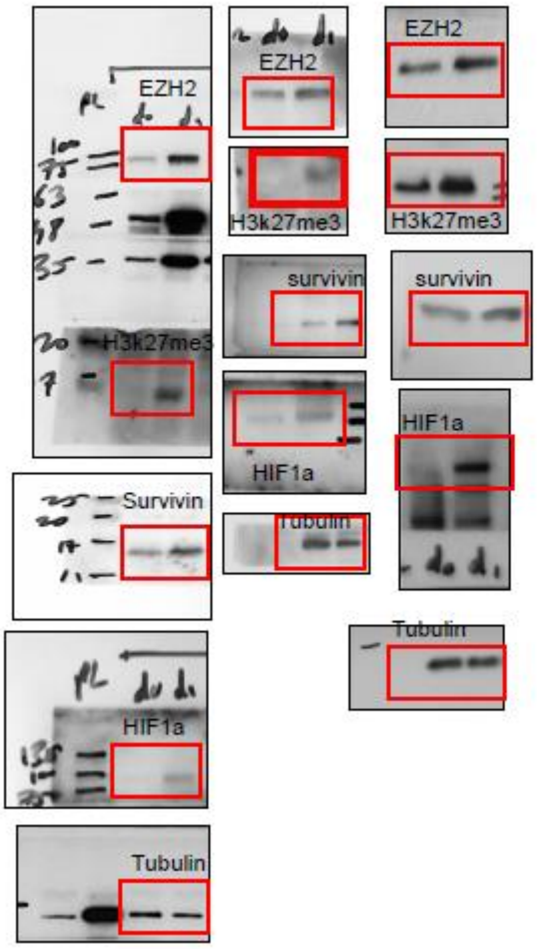

Journal of Cell Science • Supplementary information

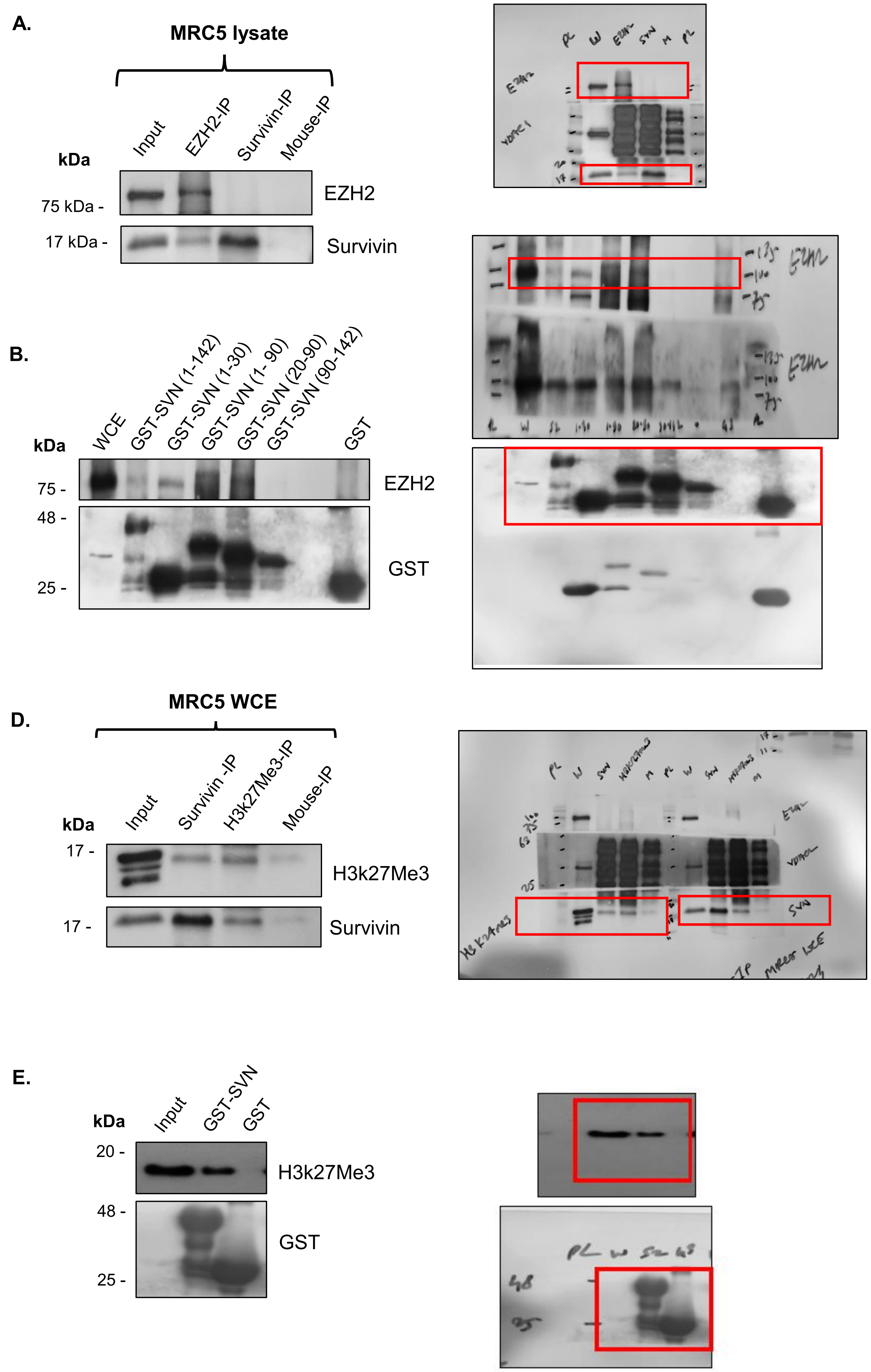

Journal of Cell Science • Supplementary information

A.

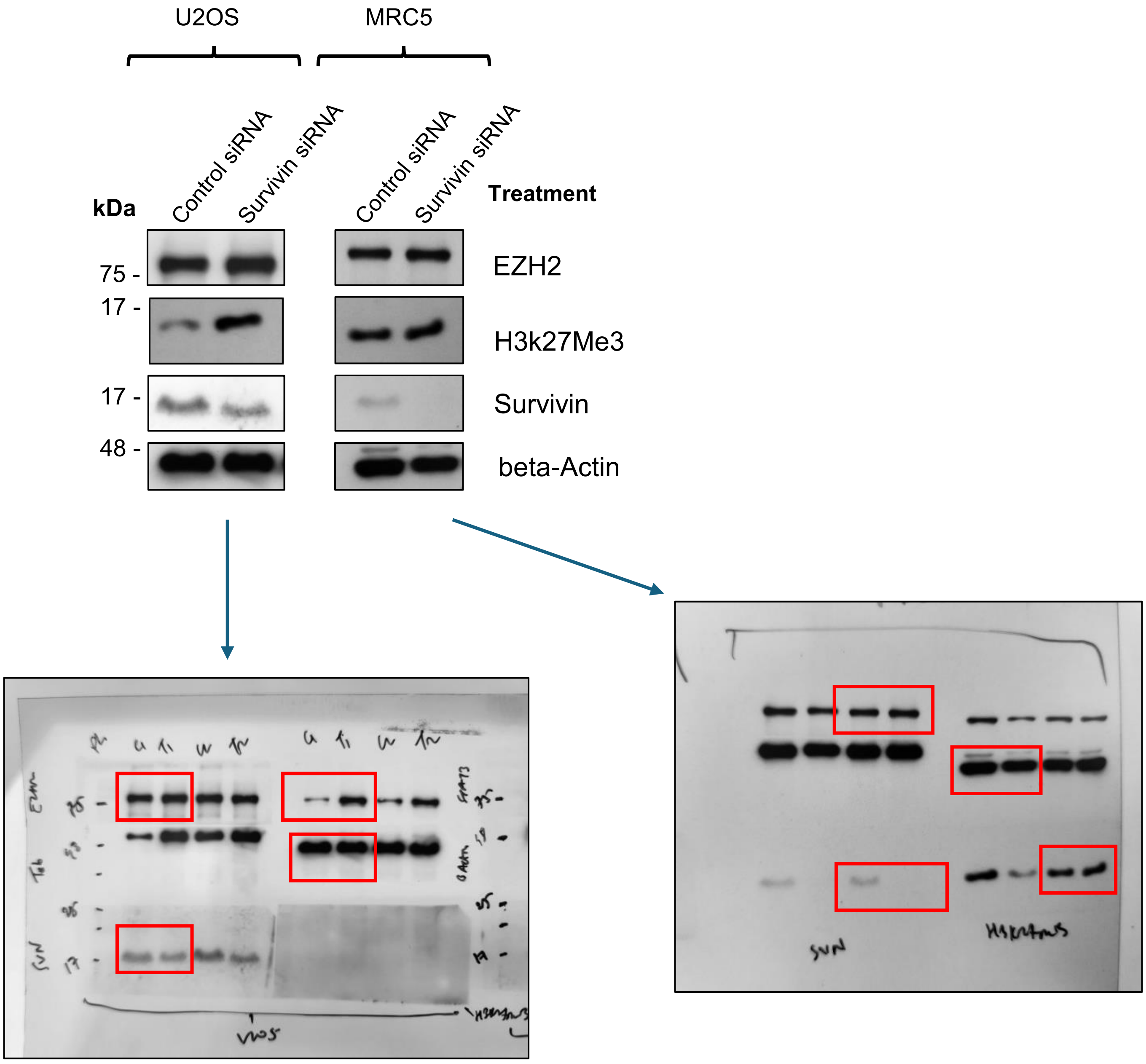
U2OS
MRC5
Control siRNA
Survivin siRNA
Control siRNA
Survivin siRNA
Treatment
kDa
75 -
17 -
17 -
48 -
EZH2
H3k27Me3
Survivin
beta-Actin

A.

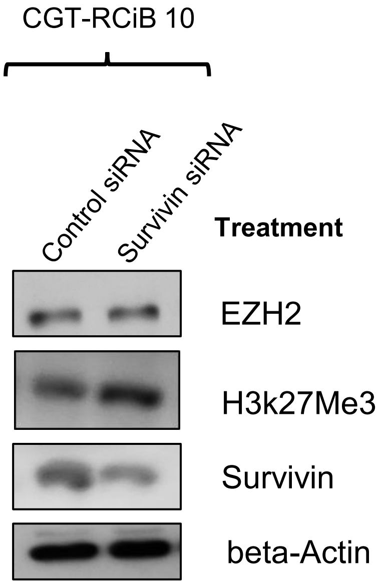

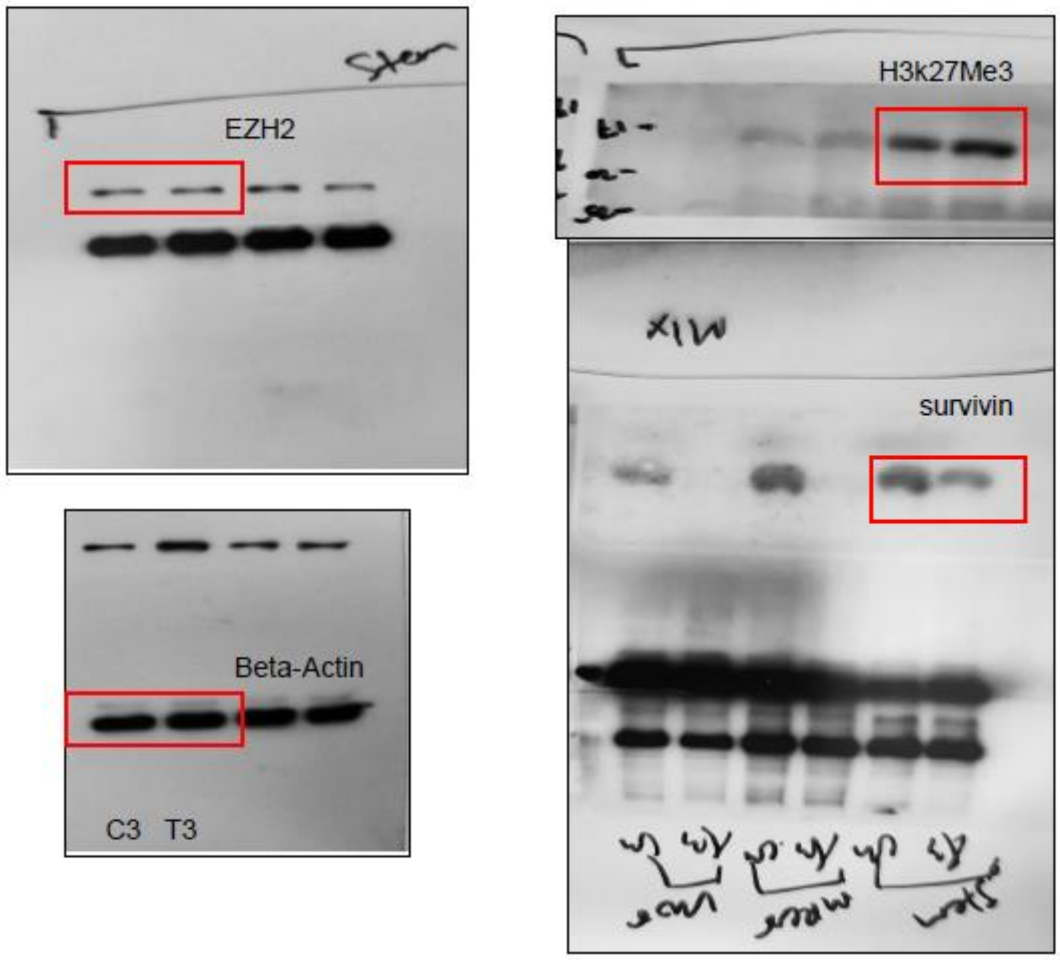

Journal of Cell Science • Supplementary information

A.

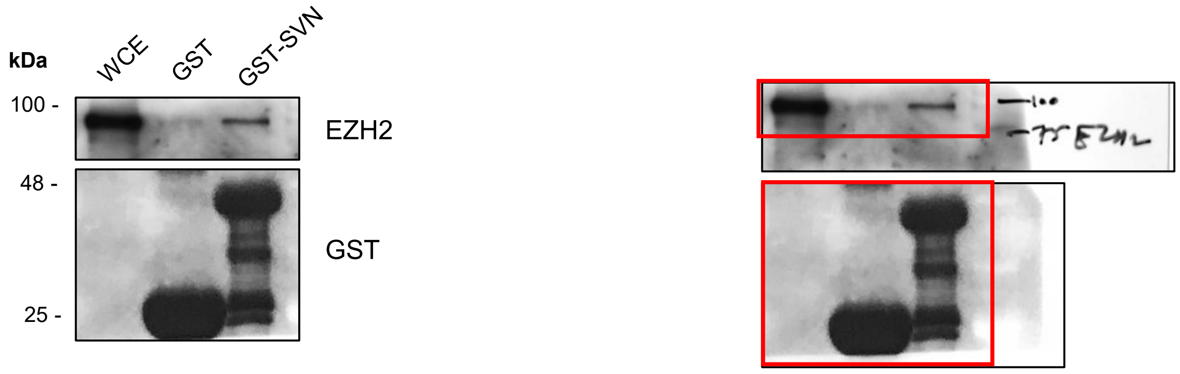

C.

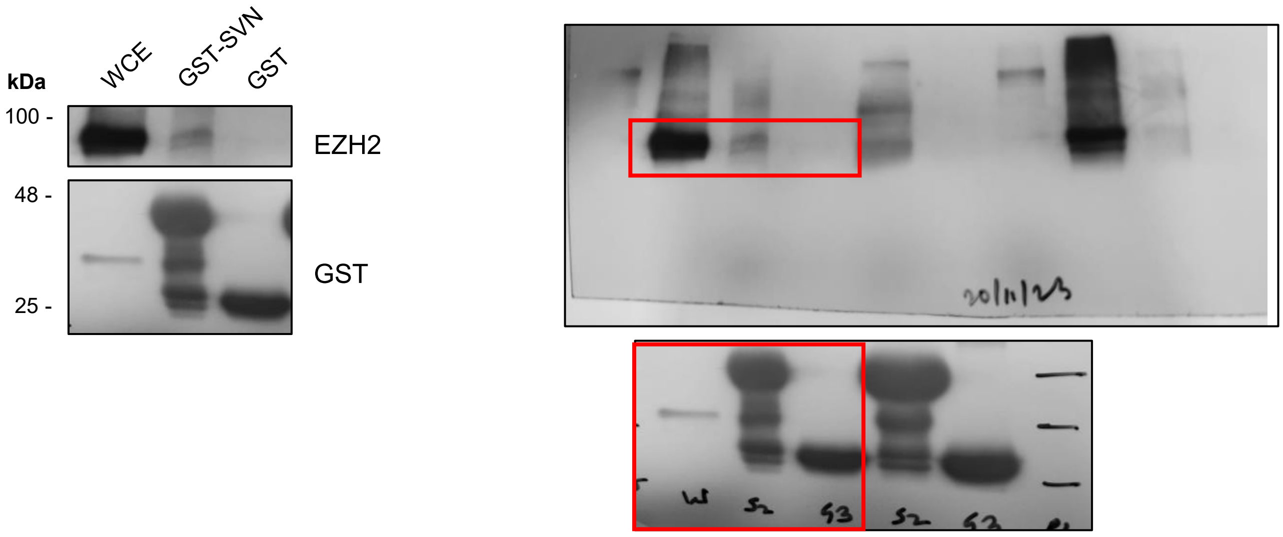

E.

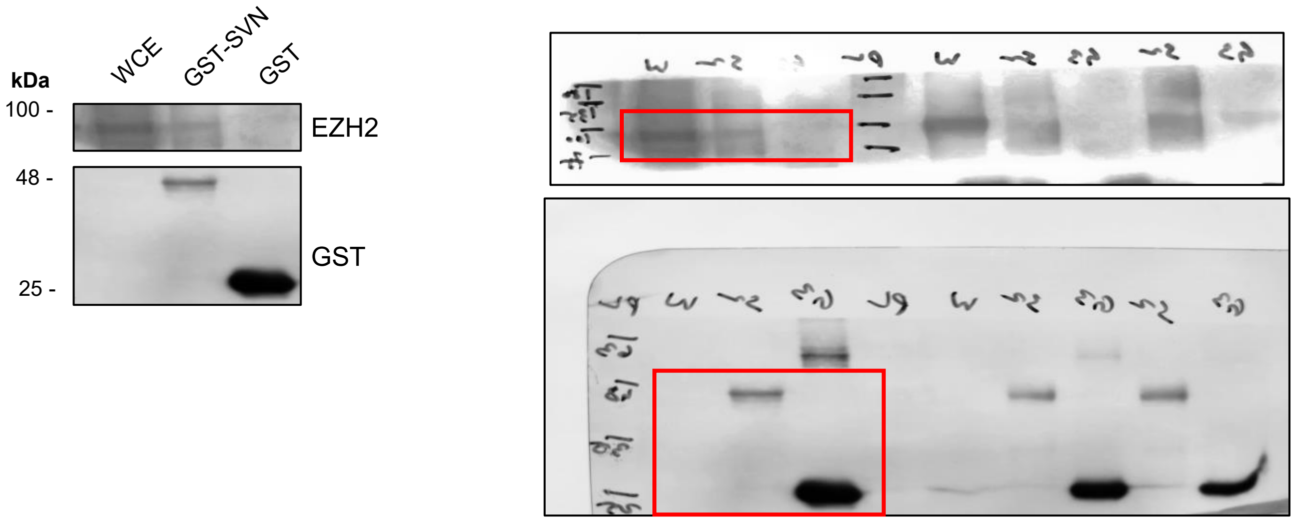

Journal of Cell Science • Supplementary information

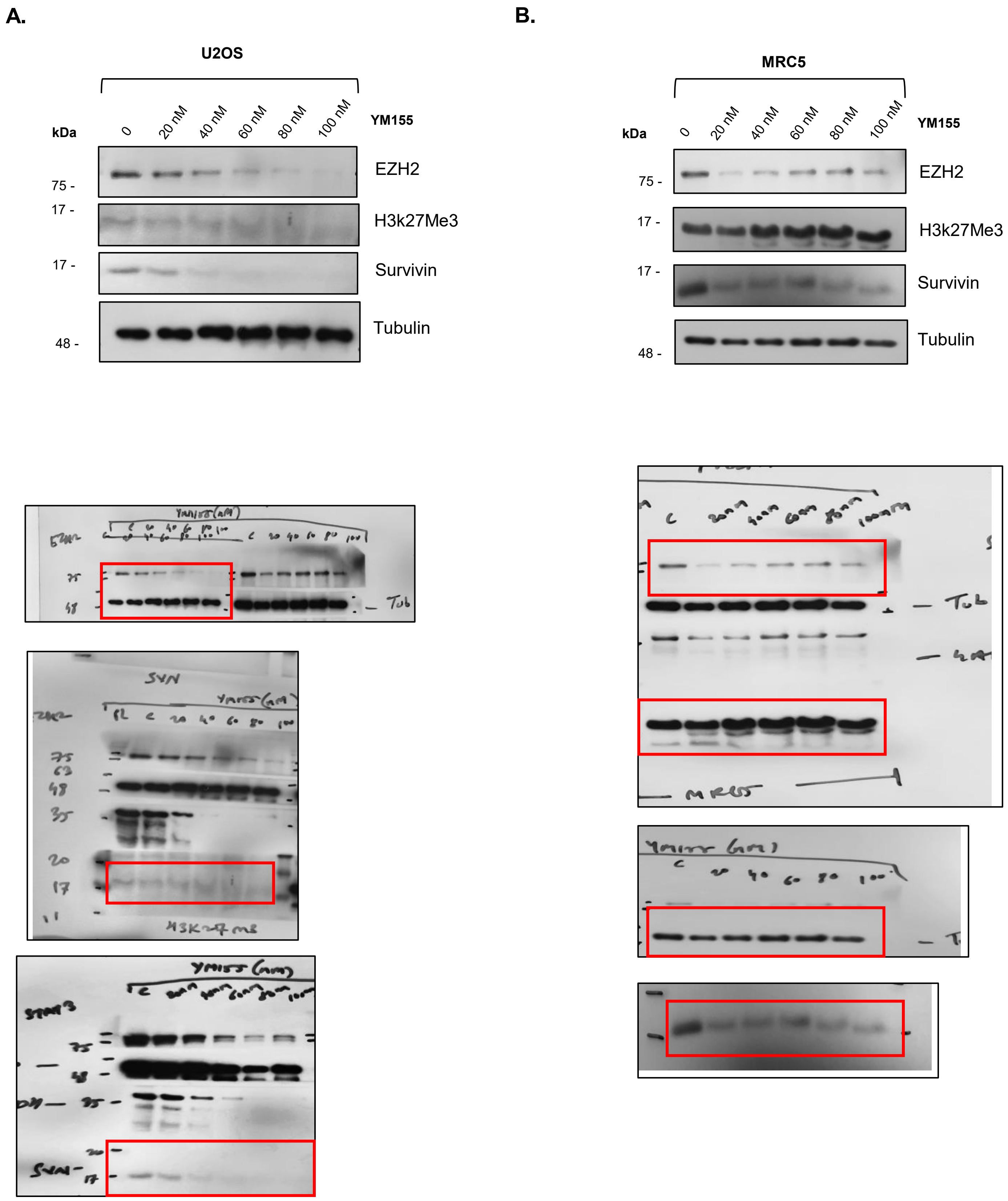

Fig. S4. Blot Transparency

Journal of Cell Science • Supplementary information
